# Bioinformatics and Raman spectroscopy-based identification of key pathways and genes enabling differentiation between acute myeloid leukemia and T cell acute lymphoblastic leukemia

**DOI:** 10.3389/fimmu.2023.1194353

**Published:** 2023-05-17

**Authors:** Haoyue Liang, Xiaodong Kong, Zhijie Cao, Haoyu Wang, Ertao Liu, Fanfan Sun, Jianwei Qi, Qiang Zhang, Yuan Zhou

**Affiliations:** ^1^ State Key Laboratory of Experimental Hematology, National Clinical Research Center for Blood Diseases, Haihe Laboratory of Cell Ecosystem, Institute of Hematology & Blood Diseases Hospital, Chinese Academy of Medical Sciences & Peking Union Medical College, Tianjin, China; ^2^ Tianjin Institutes of Health Science, Tianjin, China; ^3^ Department of Geriatrics, Tianjin Geriatrics Institute, Tianjin Medical University General Hospital, Tianjin, China

**Keywords:** AML, T-ALL, bioinformatics analysis, Raman spectroscopy, functional enrichment analysis

## Abstract

Acute myeloid leukemia (AML) and T cell acute lymphoblastic leukemia (T-ALL) are two of the most prevalent hematological malignancies diagnosed among adult leukemia patients, with both being difficult to treat and associated with high rates of recurrence and mortality. In the present study, bioinformatics approaches were used to analyze both of these types of leukemia in an effort to identify characteristic gene expression patterns that were subsequently validated *via* Raman spectroscopy. For these analyses, four Gene Expression Omnibus datasets (GSE13204, GSE51082, GSE89565, and GSE131184) pertaining to acute leukemia were downloaded, and differentially expressed genes (DEGs) were then identified through comparisons of AML and T-ALL patient samples using the R Bioconductor package. Shared DEGs were then subjected to Gene Ontology (GO) enrichment analyses and were used to establish a protein-protein interaction (PPI) network analysis. In total, 43 and 129 upregulated and downregulated DEGs were respectively identified. Enrichment analyses indicated that these DEGs were closely tied to immune function, collagen synthesis and decomposition, inflammation, the synthesis and decomposition of lipopolysaccharide, and antigen presentation. PPI network module clustering analyses further led to the identification of the top 10 significantly upregulated and downregulated genes associated with disease incidence. These key genes were then validated in patient samples *via* Raman spectroscopy, ultimately confirming the value of these genes as tools that may aid the differential diagnosis and treatment of AML and T-ALL. Overall, these results thus highlight a range of novel pathways and genes that are linked to the incidence and progression of AML and T-ALL, providing a list of important diagnostic and prognostic molecular markers that have the potential to aid in the clinical diagnosis and treatment of these devastating malignancies.

## Introduction

Acute myeloid leukemia (AML) is a form of hematological malignancy developing as a result of abnormal bone marrow cell proliferation. Affected patients exhibit the accumulation of high levels of immature, dysfunctional bone marrow cells in the blood and bone marrow. AML incidence rates rise with age and account for 15-20% of all childhood leukemia cases, in addition to being the most prevalent form of acute leukemia in adults (1). Despite the extensive morbidity and mortality associated with this form of cancer, AML patient survival rates have remained largely unchanged in recent years. Systemic chemotherapy has remained the primary treatment for AML for five decades, yet the high rates of AML tumor cell heterogeneity necessitate more robust targeted treatment strategies in order to achieve durable anticancer efficacy. The high rates of AML recurrence and associated difficulties eradicating this disease have been ascribed to leukemia stem cells (LSCs), which can proliferate indefinitely and give rise to large numbers of heterogeneous immature leukocytes in affected patients. In an effort to more reliably target AML cells, researchers have recently sought to identify patterns that are specifically activated in AML cells including the apoptosis, receptor tyrosine kinase (RTK) signaling, hedgehog (HH), mitochondrial function, DNA repair, and c-Myc signaling pathways (2). Further preclinical research focused on AML cells thus has the potential to aid efforts to more reliably diagnose and treat this form of hematological cancer.

T cell acute lymphoblastic leukemia (T-ALL) is a highly invasive form of hematological cancer resulting from the unrestrained proliferation of immature T cell progenitors that have undergone malignant transformation. T-ALL patients often exhibit a high tumor load, persistent cellular proliferation, extramedullary involvement, pleural effusion, and a large thymic mass. T-ALL accounts for 10-15% and ~25% of ALL cases in children and adults, respectively (3). While T-ALL tends to be a heterogeneous disease, cases are broadly grouped into those stemming from mutations or deletions that alter gene expression and those caused by chromosomal translocations that impact cell cycle progression or signal transduction activity (4). T-ALL tumor cells exhibit transcriptional profiles distinct from those of normal circulating blood cells, with abnormalities related to the cell cycle regulation, tumor suppressor gene expression, epigenetic regulation, RNA processing, ribosomal function, ubiquitination, Ras signal transduction, JAK-STAT signaling, PI3K-AKT-mTOR signaling, and Notch1 signaling pathways, among others. Research focused on these signaling pathways is ongoing and has the potential to inform the development of novel targeted treatments for T-ALL. the research and treatment of targeted T-ALL.

The Gene Expression Omnibus (GEO) database (http://www.ncbi.nlm.nih.gov/geo/) is an international and publically accessible database that incorporates microarray and high-throughput gene expression datasets that can be leveraged to analyze particular cancers and other diseases. GEO dataset analyses can enable the integration of data from multiple independent studies, thereby yielding a more robust dataset for clinically meaningful analyses. While heterogeneity among samples and microarray platforms can constrain to complete integration of different datasets, a range of bioinformatics strategies have been designed to facilitate large-scale cross-platform analyses of high-throughput data.

In the present study, shared differentially expressed genes (DEGs) were initially identified by comparing AML and T-ALL samples across multiple microarray and RNA-seq datasets in the GEO database, after which Gene Ontology (GO) enrichment analyses were performed and these DEGs were used to establish a protein-protein interaction (PPI) network. Raman spectroscopy was then used to verify the expression of these key genes in patient bone marrow samples, providing a robust foundation for efforts to support the differential diagnosis and treatment of AML and T-ALL.

## Materials and methods

### Data sources

Data for this study were downloaded from the NCBI GEO database. Downloaded datasets included GSE13204 (species: Homo sapiens, 716 samples, GPL570 Affymetrix HG-U133_ Plus_ 2 Array platform), GSE51082 (species: Homo sapiens, 49 samples, GPL96 Affymetrix HG-U133A Array platform), GSE89565 (species: Homo sapiens, 100 samples, GPL570 Affymetrix HG-U133_ Plus_ 2 Array platform) and GSE131184 (species: Homo sapiens, 125 samples, GPL570 Affymetrix HG-U133_ Plus_ 2 Array platform).

### Data processing and DEG identification

Expression data were downloaded in the CEL format and were processed with R v3.6.2 to correct for background expression values and normalize expression patterns, in addition to supplementing missing values. Background correction was performed using the MAS method, and data standardization was performed using quantiles.

A Gene expression matrix was used to screen for DEGs after separating samples into T-ALL and AML groups. The limma package and unpaired T-tests were used to identify DEGs based on a Benjamini and Hochberg (BG)-corrected P-value < 0.05 and a |logFC| > 1. A heatmap of these DEGs was constructed with the R pheatmap software package.

### GO enrichment analyses

GO enrichment analyses were performed using the DAVID database, assessing DEGS enriched in biological process (BP), cellular component (CC), molecular function (MF), and KEGG pathways with P < 0.05 as the significance threshold.

### PPI network construction

The STRING database (v 10.0, http://www.string-db.org/) was used to assess interactions among proteins encoded by identified DEGs using the following settings: species = Homo sapiens, PPI parameter = 0.4 (corresponding to medium confidence). The resultant PPI network was displayed using Cytoscape v3.6.0.

Node scores in the established network were analyzed based on the degree centrality topological parameter, with a higher score indicating that a given node is more important in the overall network such that it is more likely to represent a key node. The possible functions of the top 10 genes identified when comparing the T-ALL and AML groups were analyzed in further detail.

### Sub-network module analyses

In a complex biological system, individual proteins function through complex regulatory interactions with other targets rather than functioning in isolation, However, proteins included in a given module often engage in similar or related functions. Accordingly, the Cytoscape ClusterONE plug-in was used to analyze significantly clustered modules within established PPI networks.

### Sample collection

Samples of bone marrow were collected from acute leukemia patients (8 male, 2 female; 0-58 years of age) hospitalized in the Blood Diseases Hospital of the Chinese Academy of Medical Sciences (Institute of Hematology of the Chinese Academy of Medical Sciences) between January and June of 2016. All of these patients had undergone bone marrow puncture, bone marrow cell morphology, flow cytometry, histochemistry, chromosome, gene fusion, and electron microscopy analyses. Patients had been diagnosed in accordance with the results of histochemistry, morphology, and other examinations, with confirmation based on FAB. These patients included 3 T-ALL patients and 7 AML patients, including 2 patients with AML M7 and 1 each with AML M1, M2, M3, M5, and M6. The Ethics Committee of the Blood Diseases Hospital of the Chinese Academy of Medical Sciences provided approval for this study (KT2020016-EC-2). All included patients underwent routine serum biochemical testing that was obtained from patient medical records.

### Raman spectroscopy

In total, 5 μL bone marrow supernatant samples were applied to quartz slides, followed by analysis with a confocal Raman spectrometer XploRA Raman microscope. A 785 nm excitation laser was utilized with a 40x objective lens and an output power of 10 mW. Samples were fixed to a three-dimensional XYZ platform. Imaging was achieved with a 40x 0.6 NA Nikon lens, with a spot size of 2x2 μm and a measurement range of 600-1800 cm^-1^. In total, 6 sites were measured per sample at a resolution of 1 cm^-1^. The Raman spectrum of quartz was additionally analyzed to assess the background signal. Data smoothing, background signal removal, and baseline correction were performed using Labspec 6. Spectral normalization was achieved using 1450 cm^-1^ Raman peaks as internal standards.

### Raman spectrum data analyses and diagnostic model development

The Raman spectrum data from acute leukemia patients generated above were subjected to OPLS-DA analyses performed using SIMCA14.1. OPLS model performance was evaluated based on the R^2^ and Q^2^ goodness of fit parameters. Model resampling was performed 200 times through the random modulation of the y matrix to validate model results. Cluster analyses and receiver operating characteristic (ROC) curve analyses were performed. Significant Raman peaks in the classification model were identified as potential biomarkers through a V+S analytical approach. Briefly, peaks with a variable importance in projection (VIP) value > 1.5 and a correlation coefficient (distance from the center of the V+S diagram) in the same range as a potential biomarker were selected, with those biomarker candidates exhibiting a P-value < 0.05 being considered significant. Data processing was performed with the Origin software, while statistical analyses were conducted using SPSS 20.0 (IBM, USA), and figures were constructed using GraphPad Prism 5.

## Results

### Identification of genes differentially expressed in AML and T-ALL patient samples

After initial preprocessing of data from four GEO datasets (GSE13204, GSE51082, GSE89565, and GSE131184), 54675, 22283, 54675, and 54675 probes were identified, respectively, with average values being calculated when multiple probes corresponded to a given gene symbol. Following probe annotation, 20174, 12402, 20174, and 20174 genes were obtained from these respective datasets, of which 940, 565, 834, and 879 were significantly differentially expressed between AML and T-ALL samples (**Supplementary Tables 5-12**). These included 348, 236, 268, and 277 significantly upregulated DEGs as well as 592, 329, 566, and 602 significantly downregulated DEGs.

**Figure 1:**
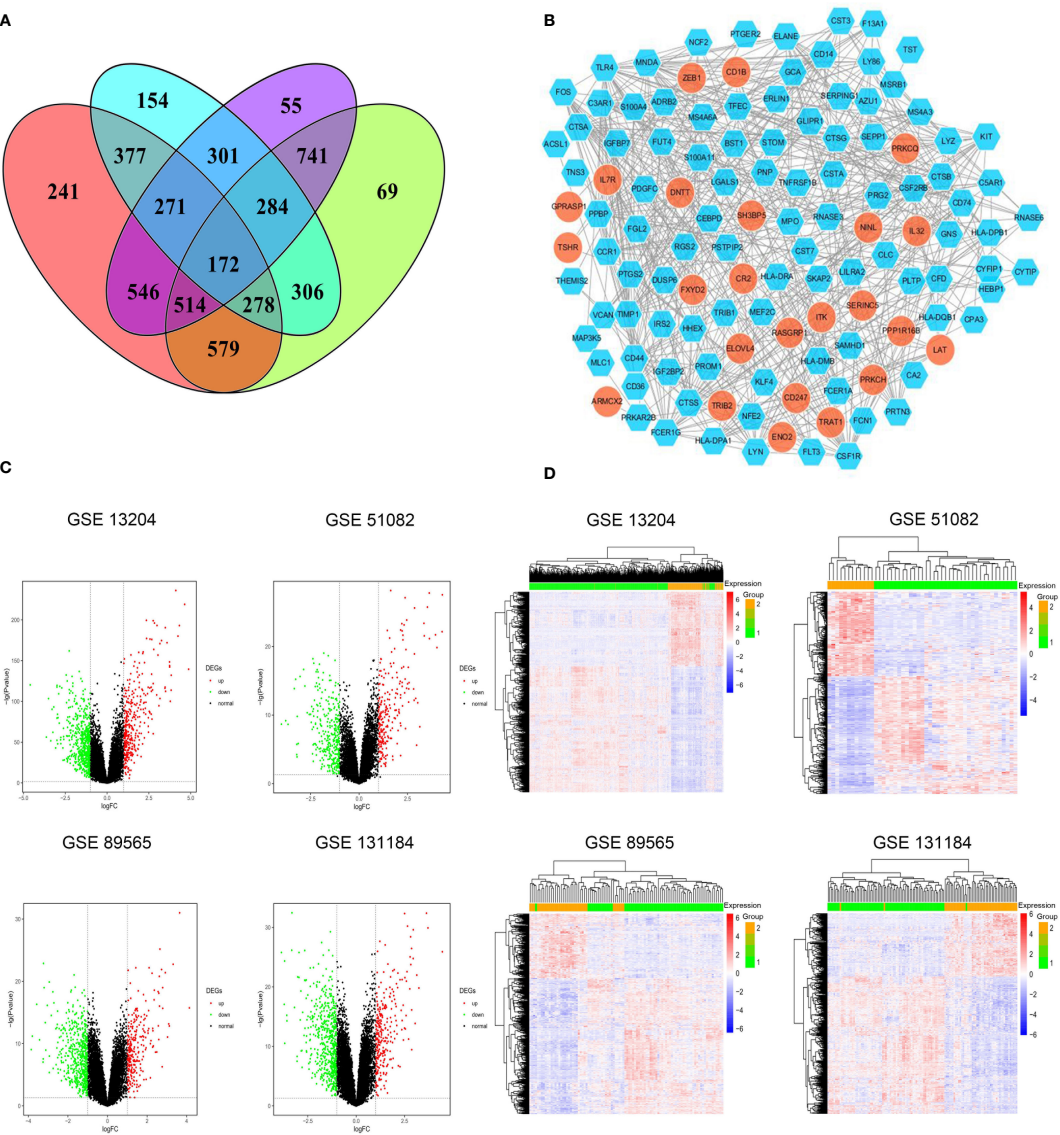
**(A)** Identification of significant DEGs between T-ALL and AML patient samples in the GSE13204, GSE51082, GSE89565, and GSE131184 datasets. **(B)** A protein-protein interaction network for the identified DEGs. **(C)** Volcano plots highlighting gene expression in each dataset, with individual points corresponding to specific genes and red and green colors indicating significantly upregulated and downregulated genes, respectively (P < 0.05, FC > 1). **(D)** Heatmaps were generated to display DEG expression profiles in individual patient samples.

DEGs shared among these four datasets are shown in **Figures 1A, B**. Of the identified DEGs, 43 significantly upregulated genes and 129 significantly downregulated genes were shared across all four independent datasets. These upregulated (log2FC > 1, P < 0.05) and downregulated (log2FC < -1, P < 0.05) genes are listed in **Supplementary Tables 3, 4**, respectively. Distributions of gene expression between T-ALL and AML patient samples in these different datasets were further displayed using Volcano plots (**Figure 1C**), with red and green dots respectively representing genes that were significantly upregulated and downregulated. Heatmaps were also generated to display patterns of DEG expression in these four datasets, with columns corresponding to samples and rows corresponding to genes while coloration corresponds to the relative expression of the indicated gene in the indicated sample. In the DEG heatmap, the red and blue colors indicate the upregulation and downregulation, respectively, of DEGs, while the yellow and green colors indicate T-ALL and AML samples, respectively (**Figure 1D**).

### Functional enrichment analyses

**Figure 2:**
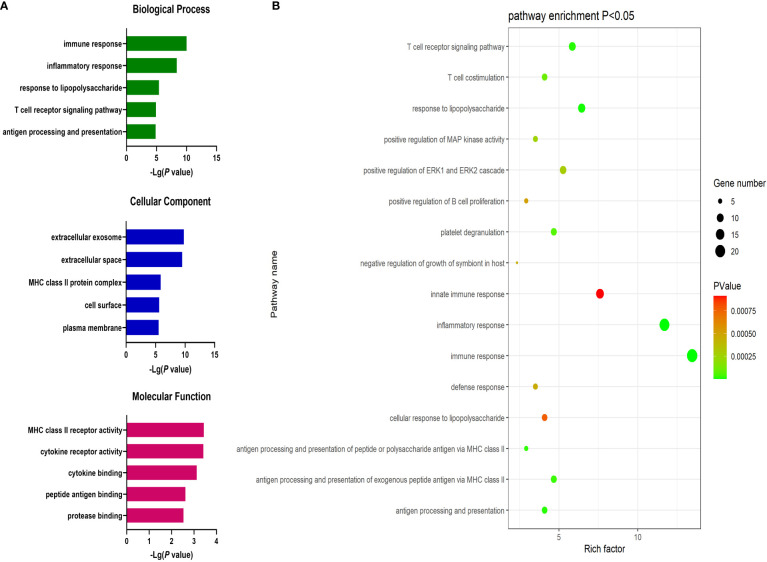
**(A)** GO enrichment analysis results for DEGs between AML and T-ALL patient samples. **(B)** Enrichment results for the pathways most closely associated with the differentiation between AML and T-ALL.

Next, the DAVID database was used to conduct GO enrichment analyses for the identified DEGs. These genes were enriched in 87 GO-BP pathways that were primarily related to the immune response, inflammation, antigen processing and presentation, lipopolysaccharide responses, and the T cell receptor signaling pathway. These genes were further enriched in 30 GO-CC terms including the extracellular exosomes, extracellular spaces MHC class II protein complex, and cell surface terms. These DEGs were also enriched in 19 GO-MF terms including the cytokine receptor activity, MHC class II receptor activity, cytokine binding, peptide antigen binding, and protein binding pathways (**Figure 2**).

**Figure 3:**
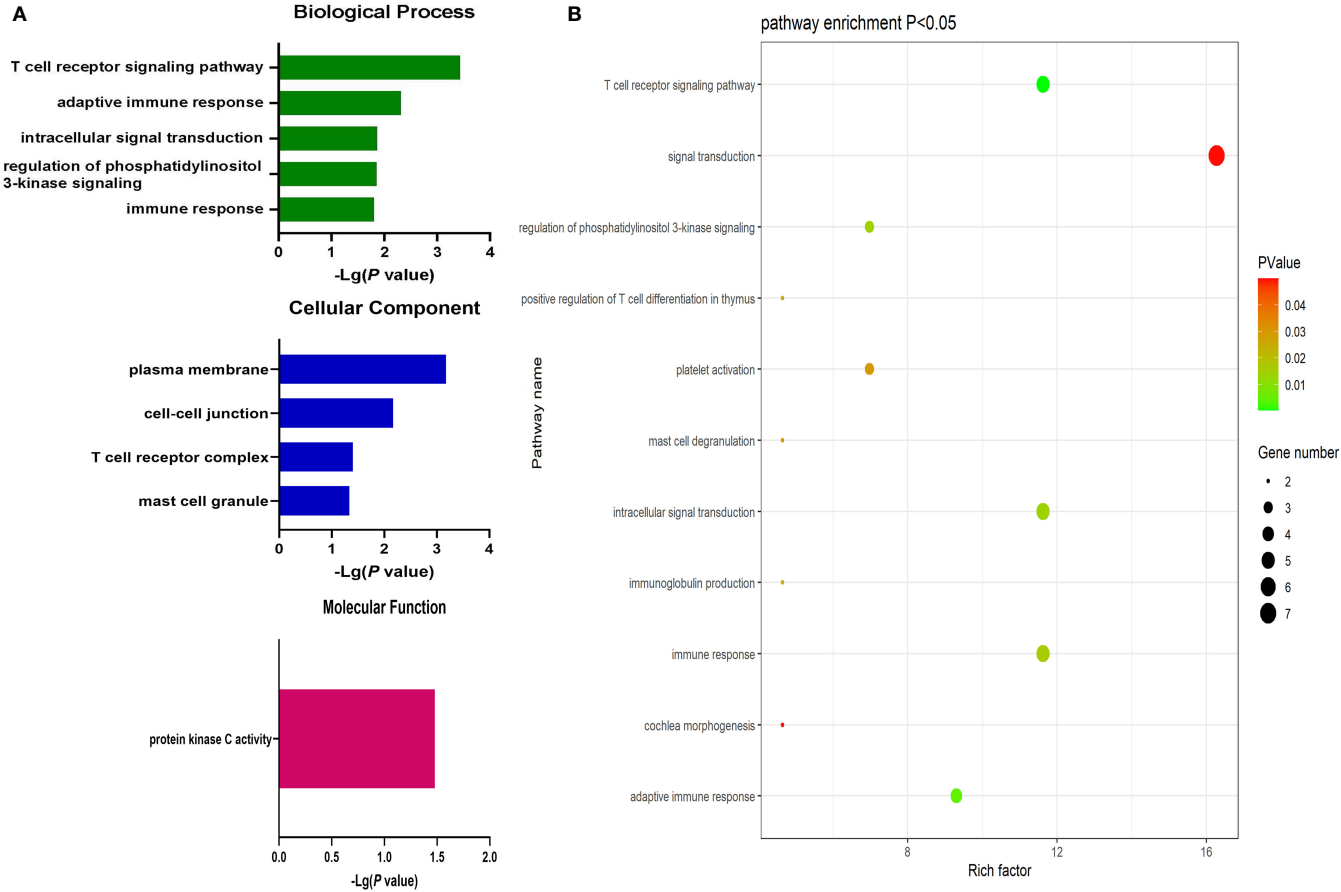
**(A)** GO enrichment analysis results for upregulated DEGs between AML and T-ALL patient samples. **(B)** Enrichment results for the pathways most closely associated with the differentiation between AML and T-ALL based on the identified upregulated DEGs.

**Figure 4:**
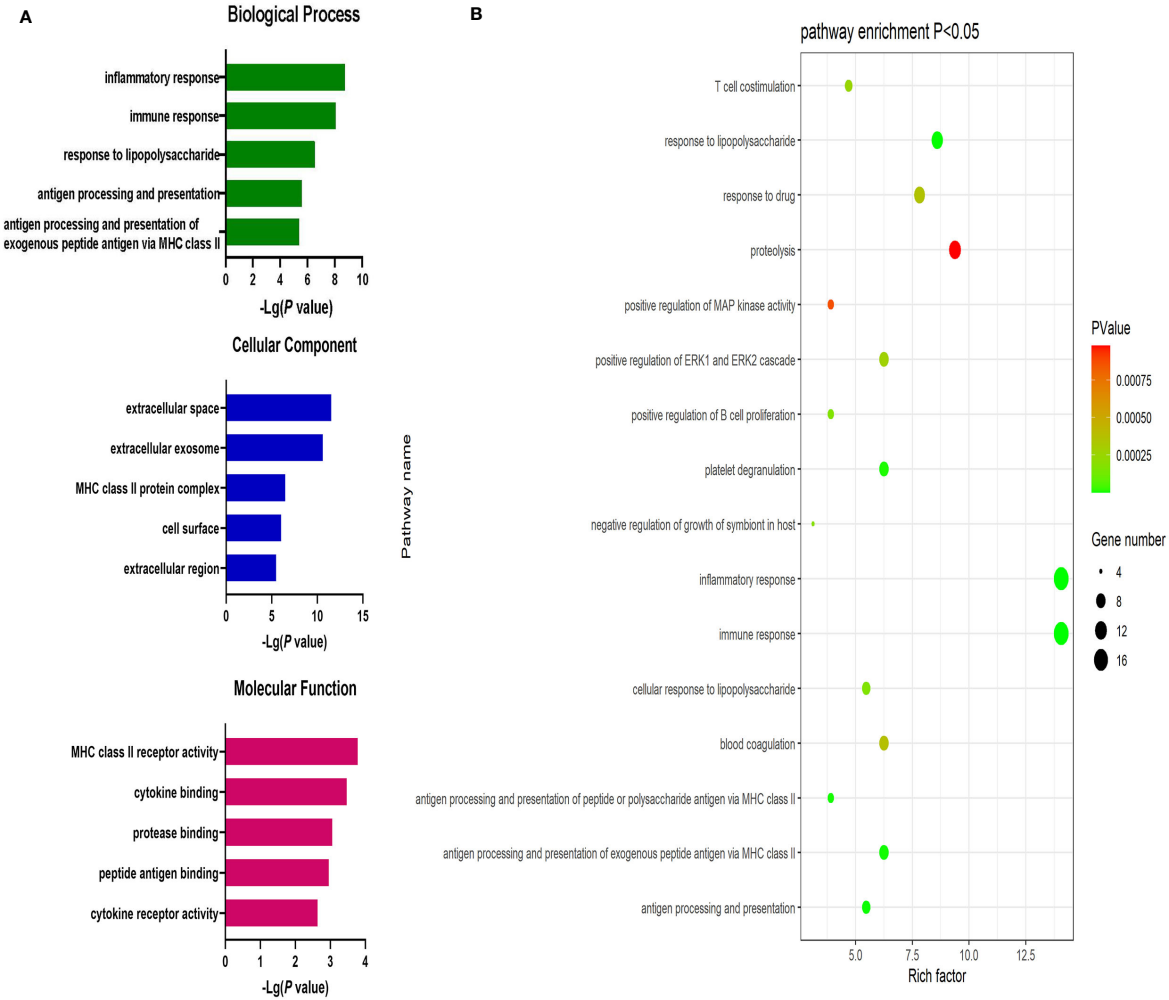
**(A)** GO enrichment analysis results for downregulated DEGs between AML and T-ALL patient samples. **(B)** Enrichment results for the pathways most closely associated with the differentiation between AML and T-ALL based on the identified downregulated DEGs.

Significantly upregulated DEGs were enriched in 11 GO-BP terms including the T cell receptor signaling pathway, adaptive immune response, and intracellular signal transduction pathways. They were also enriched in a single GO-MF pathway (protein kinase C activity) and four GO-CC terms including the plasma membrane, cell-cell junction, T cell receptor complex, and mast cell granule terms (**Figure 3**). Moreover, significantly downregulated DEGs were enriched in 84 GO-BP terms that were primarily associated with the immune response, inflammation, antigen processing and presentation, and lipopolysaccharide responses. These downregulated genes were also enriched in 30 GO-CC terms including the extracellular space and extracellular exosome terms, as well as 17 GO-MF terms including the MHC class II receptor activity, cytokine binding, protein binding, peptide antigen binding, and cytokine receptor activity pathways (**Figure 4**). The top 5 most significantly enriched GO terms in each of these categories are presented in **Supplementary Tables 13-15**.

### PPI network and network clustering module analyses

The identified DEGs were used to establish a PPI network consisting of 122 nodes and 512 interaction pairs (**Figure 1B**). Those nodes with high topological degree values were identified as key nodes in the overall network, and corresponding degree values for the top 10 genes are shown in **Supplementary Table 16**. The 10 key DEGS screened from this network were TLR4, MPO, MNDA, CSF1R, CD44, C3AR1, FCER1G, CTSS, LYN, and FOS. The majority of these DEGs were significantly downregulated, indicating that they were significantly overexpressed in AML.

**Figure 5:**
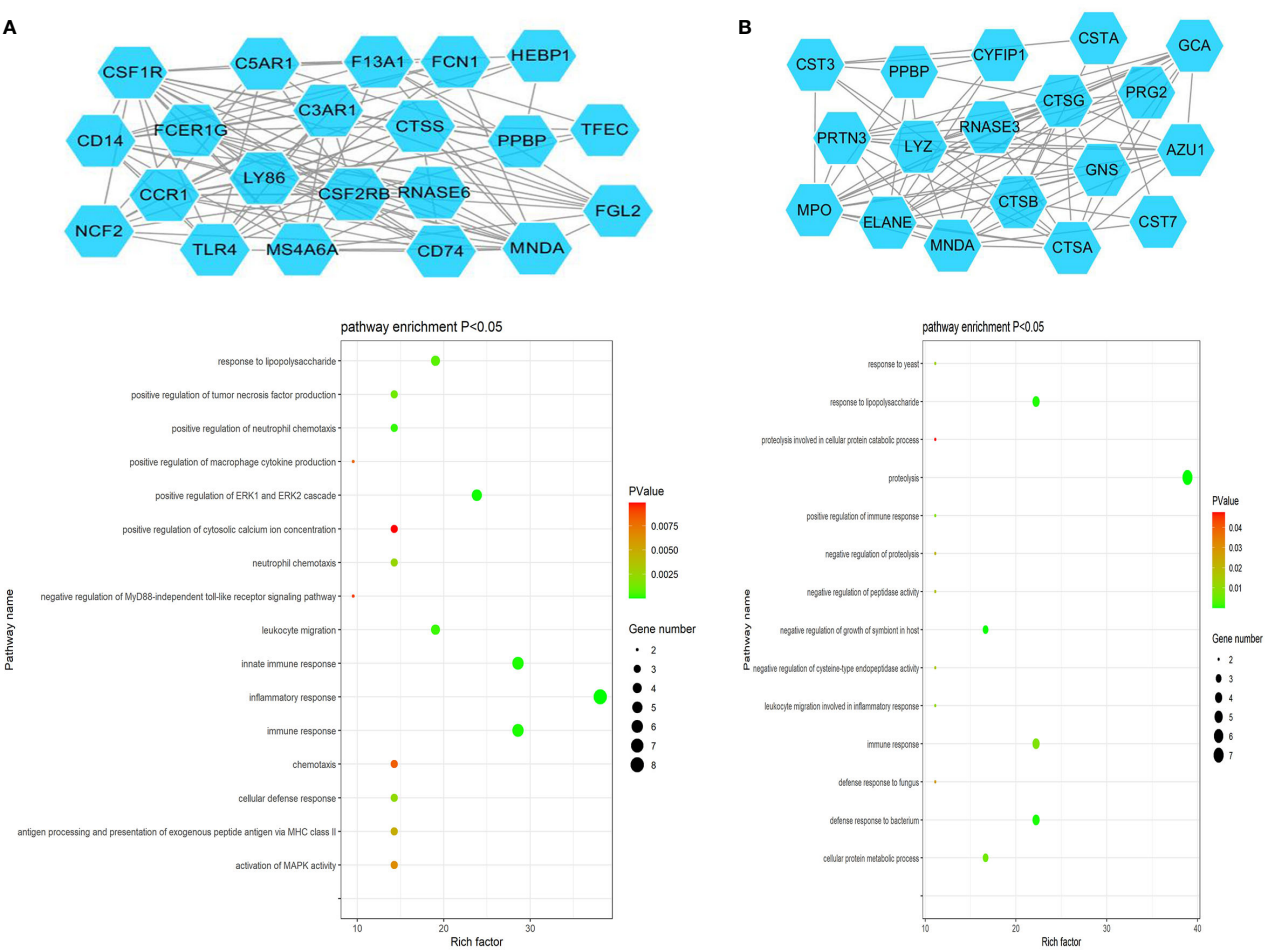
PPI Module 1 **(A)** and Module 2 **(B)** identified through analysis of the interactions of DEGs between AML and T-ALL patient samples, including information on the functions and regulation of these genes.

**Figure 6:**
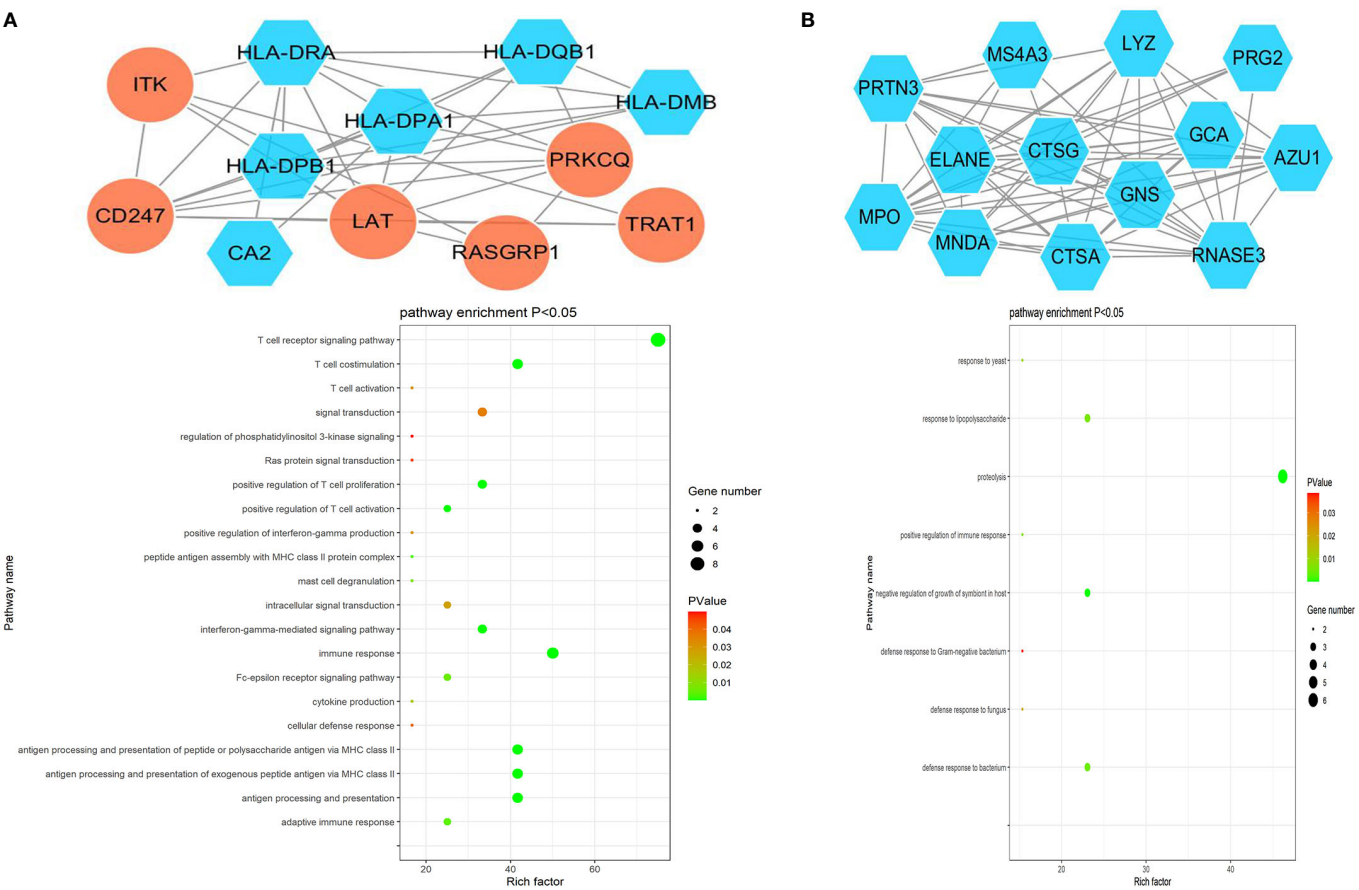
PPI Module 3 **(A)** and Module 4 **(B)** identified through analysis of the interactions of DEGs between AML and T-ALL patient samples, including information on the functions and regulation of these genes.

**Figure 7:**
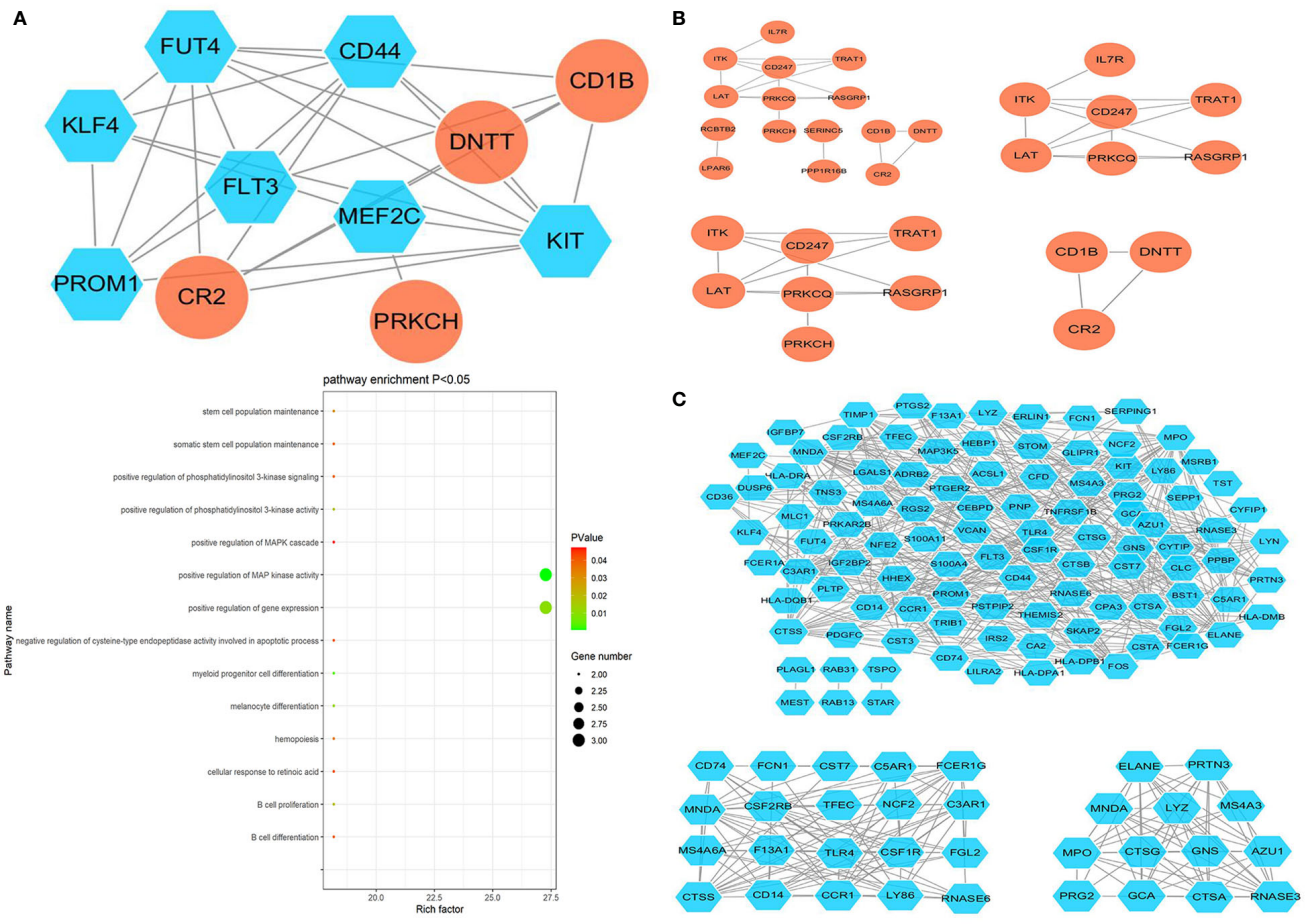
**(A)** PPI Module 5 identified through analysis of the interactions of DEGs between AML and T-ALL patient samples, including information on the functions and regulation of these genes. **(B)** Four modules identified when specifically analyzing upregulated DEGs between AML and T-ALL samples. **(C)** Three modules identified when specifically analyzing downregulated DEGs between AML and T-ALL samples.

The ClusterONE Cytoscape plug-in was further used to analyze DEGs, leading to the identification of five significant module clusters consisting of 21, 18, 12, 13, and 11 nodes, and 106, 79, 36, 64, and 28 interaction pairs (**Figures 5**–**7**). Module 1 was primarily associated with the inflammatory response, immediate response, and the positive regulation of ERK1/2 signaling (**Figure 5A**). Module 2 was primarily associated with proteolysis, the negative regulation of growth, and bacterial defense responses (**Figure 5B**). Module 3 was primarily associated with T cell receptor signaling and antigen processing and MHC class II presentation (**Figure 6A**). Module 4 was primarily associated with proteolysis, the negative regulation of growth, and bacterial defense responses (**Figure 6B**). Module 5 was primarily associated with the positive regulation of MAPK activity, myeloid cell differentiation, and the positive regulation of gene expression (**Figure 7A**).

When the upregulated DEGs were specifically subjected to module analyses, three significant modules comprised of 7, 7, and 3 nodes, and 12, 12, and 3 interaction pairs were established (**Figure 7B**). Similarly, two significant modules were identified when analyzing downregulated DEGs consisting of 20 and 13 nodes, and 99 and 64 interaction pairs, respectively (**Figure 7C**).

### Raman spectrum analyses of bone marrow samples from acute leukemia patients

**Figure 8:**
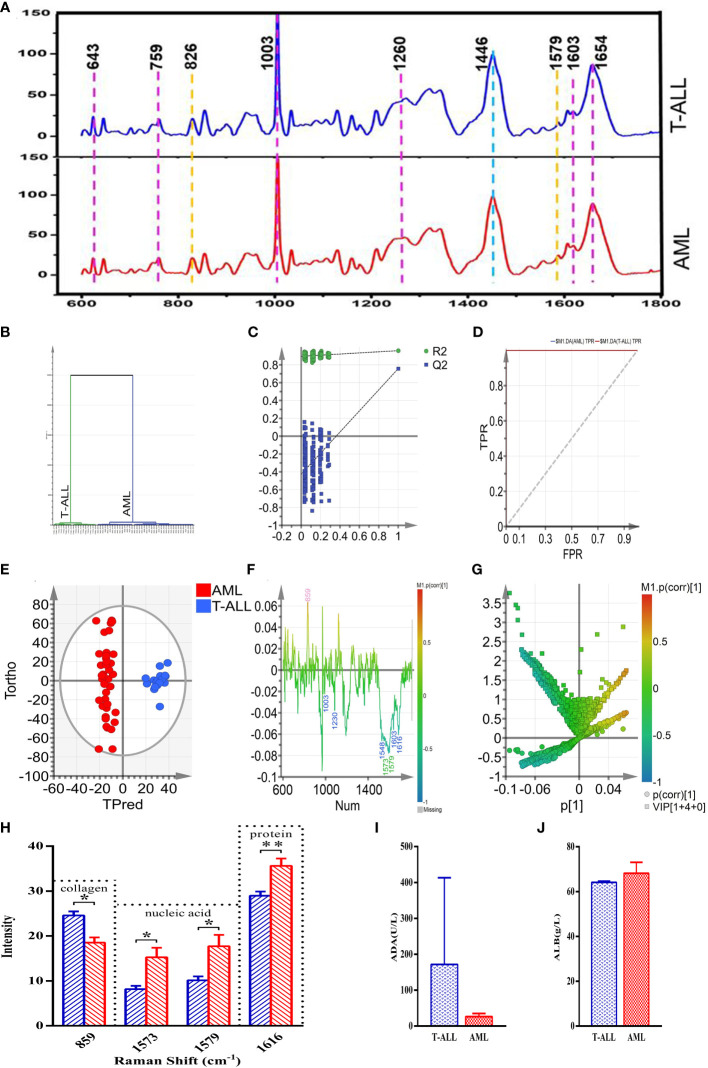
**(A)** (from bottom to top) the average spectra of the AML and T-ALL groups. **(B)** OPLS-DA cluster plot for the comparison of AML and T-ALL groups. **(C)** OPLS-DA permutation plots for the comparison of AML and T-ALL groups. **(D)** ROC curves for the OPLS-DA model when comparing the AML and T-ALL groups, AUC (AML) =1, AUC (T-ALL) =1. **(E)** OPLS-DA score plot for discrimination between the AML and T-ALL groups drawn with Hotelling’s 95% confidence ellipse, R^2^X = 0.548, R^2^Y = 0.959, and Q^2 = ^0.756. **(F)** Loading line plot corresponding to discrimination between the AML and T-ALL groups. **(G)** V+S plot corresponding to discrimination between the AML and T-ALL groups. The red, pink, green, blue, and yellow numbers are associated with cholesterol, collagen, nucleic acids, proteins, and carbohydrates, respectively. **(H)** Statistical analysis of potential biomarkers identified from OPLS-DA models comparing AML and T-ALL samples. **(I)** Comparisons of ADA between the AML and T-ALL groups. **(J)** Comparisons of ALB between the AML and T-ALL groups. *P < 0.05, **P < 0.01.

In an effort to validate the above results, 18 and 40 Raman characteristic spectra were respectively obtained from 3 T-ALL and 7 AML patients, including 5 spectra from AML-M1 patients, 6 spectra from AML-M2 patients, 6 spectra from AML-M3 patients, 6 spectra from AML-M5 patients, 6 spectra from AML-M6 patients, and 11 spectra from AML-M7 patients. Peak assignments for these Raman spectra are shown in **Supplementary Table 1** for the analyzed bone marrow supernatants in the 600~1800 cm^-1^ range. These samples exhibited similar morphological characteristics, and the resultant spectra can thus effectively reflect the content of the bone marrow supernatants from these different subsets of acute leukemia patients (**Figure 8A**). Based on these spectral patterns alone, however, it is not possible to differentiate between T-ALL and AML patients. Accordingly, multivariate statistical methods are necessary to establish reliable classification models.

### Development of a method for the Raman spectroscopy-based classification of acute leukemia patient samples

The 58 characteristic Raman spectra obtained from these AML and T-ALL patients were used as two separate sets of data to conduct a supervised orthogonal partial-least-squares discriminant analysis (OPLS-DA). This OPLS-DA model was able to distinguish between AML and T-ALL bone marrow samples with 100% accuracy based on a cluster analysis approach (**Figure 8B**). Permutation analyses revealed a negative Q^2^ intercept on the Y-axis consistent with the absence of overfitting (**Figure 8C**). ROC curve analyses of this model yielded area under the curve values of 1 for both AML and T-ALL when conducting this tumor-type comparison (**Figure 8D**), suggesting a high degree of reliable discriminative performance.

### Potential biomarker identification

To better screen for biomarkers capable of differentiating between AML and T-ALL patient bone marrow supernatant samples, this OPLS-DA model was used for further analyses. In the OPLS-DA score plot shown in **Figure 8E**, the x-axis and y-axis respectively correspond to the score values for the main orthogonal signal correction (OSC) components and the score values for the orthogonal OSC components, with differences between samples in a given group being evident based on the direction of the ordinate. The T-ALL and AML samples were effectively separated from one another in the generated score plot, confirming the ability of a supervised OPLS-DA method to differentiate between these two forms of cancer. The OPLS-DA loading plot was utilized for a preliminary analysis of those Raman peaks that contribute to this AML vs. T-ALL classification model (**Figure 8F**), with red, pink, green, blue, and yellow peak numbers respectively relating to cholesterol, collagen, nucleic acids, proteins, and carbohydrates. Correlations were evident between the loading and score plots pertaining to the levels of different materials in samples from these two patient groups. The intensity of the peak corresponding to collagen (859 cm^-1^) in the T-ALL group was elevated as compared to the AML group, whereas the intensity values for peaks corresponding to proteins (1003, 1230, 1548, 1603, and 1616 cm^-1^) and nucleic acids (1573 and 1579 cm^-1^) were lower than those in the AML group (**Figure 8F**).

An OPLS-DA V+S plot integrating VIP and correlation coefficient parameters corresponding to the contributions of peak position to the classification model is shown in **Figure 8G**. Individual points in this figure represent peak positions, with red corresponding to a higher correlation coefficient indicative of a greater contribution of the corresponding peak to the classification model. In contrast, blue indicates a weaker correlation coefficient and a less significant contribution of that peak position to the established classification model. The further a given peak position point is from the center of the established V+S plot, the greater its contribution to the established classification model. To better evaluate the contributions of peak positions to this classification model, the V+S plot was used for biomarker screening and significance analyses of characteristic peak positions capable of effectively discriminating between AML and T-ALL samples (**Figure 8G**). Corresponding VIP values for the Raman peak positions in this classification model enable the assessment of the contributions of the corresponding peaks for the established model, with those peaks exhibiting a VIP > 1.5 being screened as possible biomarkers. Based on analyses of factors including VIP values, correlation coefficients, load, and distance from the center of the V+S plot, the key peaks contributing to T-ALL vs. AML sample classification were thus identified. Those Raman peaks that did not exhibit any significant differences in these analyses were excluded from further biomarker analyses.

### Biomarker validation

To validate the candidate biomarkers identified when differentiating between AML and T-ALL patient bone marrow samples, statistical analyses of all Raman data involved in the established model were conducted (**Figure 8H**). The intensity of the peak position corresponding to collagen (859 cm^-1^) was significantly higher in the T-ALL group as compared to the AML group, whereas the intensity values for the peak positions corresponding to nucleic acids (1573, 1579 cm^-1^) and proteins (1616 cm^-1^) were significantly lower in the AML group (**Figure 8H**). These findings are consistent with the results of serum biochemical analyses (**Figure 8H**). T-ALL is associated with the excessive proliferation of leukemic cells, significantly impacting the hematopoietic microenvironment and resulting in pronounced changes in the degradation and synthesis of collagen in the extracellular matrix. T-ALL patients are more susceptible to liver damage, resulting in elevated serum ADA levels and decreased nucleic acid content (**Figure 8H**). The high rates of metabolic activity observed for bone marrow cells in T-ALL patients also contribute to the consumption of large quantities of amino acids and proteins such that the total protein levels in the peripheral blood of these patients were decreased as compared to patients with AML (**Figure 8J**). Conversely, AML patients exhibited higher levels of serum total protein relative to T-ALL patients while their ADA content was decreased (**Figure 8I**). The better prognosis observed for AML patients may be related to these biochemical results, suggesting that analyses of serum total protein content and ADA levels may offer value in the prognostic evaluation of AML and T-ALL patients.

## Discussion

AML and T-ALL are highly prevalent forms of hematologic malignancies. Despite marked advances in the treatment of these diseases using a range of antibody-drug conjugates, small molecule kinase inhibitors, hypomethylating agents, and allogeneic hematopoietic stem cell transplantation (HSCT), over half of patients do not meet the requirements of pre-intensive chemotherapy. HSCT and chemotherapy-induced remission can also lead to a range of severe treatment-related complications, and both recurrence and mortality rates are high in these cancer patients (1). As such, further research is needed to improve the diagnosis, treatment, and prognosis of these forms of hematological cancer.

In the present study, four independent GEO datasets comparing AML and T-ALL patient samples were screened, leading to the identification of 172 shared DEGs (43 upregulated, 129 downregulated). Functional analyses revealed these DEGs to be related to processes including collagen catabolism, cell adhesion, and extracellular matrix decomposition. Bioinformatics and PPI network module analyses further led to the identification of 10 key DEGs including TLR4, MPO, MNDA, CSF1R, CD44, C3AR1, FCER1G, CTSS, LYN, and FOS. The majority of these genes were expressed at significantly higher levels in AML samples relative to T-ALL samples.

Of the DEGs identified in this study, 43 were significantly overexpressed in T-ALL patient tumor cells. T-ALL tumors are derived from malignant T cell precursor transformation, and harbor diverse genetic abnormalities. Broadly speaking, these tumors generally develop as the result of chromosomal translocation or deletion/mutation events that impact cell cycle progression or signal transduction activity. Chromosomal translocations have been confirmed to be present in the precursor cells of roughly half of all T-ALL patients. These chromosomal changes generally coincide with T cell receptor (TCR) rearrangement that drives the overexpression of proto-oncogenes such as TLX1 (HOX11), MEF2C, HOXA, LMO1, LMO2, and TAL1 (5, 6). LMO1, LMO2, and TAL1 rearrangements are classified as TCRD rearrangements that impact 9% of patients, while TCRB or TCRD TLX1 rearrangements affect 10% of patients, and TCRB HOXA rearrangements affect 5% of patients (7). Other patients exhibit rearrangements affecting transcription factor genes including PCIALM-MLLT10, STIL-TAL1, TLX3-BCL11B, and NUP214-ABL1, which respectively impact 8%, 20%, 15%, and < 5% of patients, respectively (5, 6). Abnormal gene fusion events such as EML-ABL1 and SET-NUP214 fusions can also arise in some cases. MLL gene rearrangement affects 5-10% of all patients with T-ALL, but the ability of these rearrangements to independently predict patient minimum residual disease (MRD) outcomes at the end of consolidation therapy has yet to be confirmed (8). Genomic analyses of 264 T-ALL patient samples, including full exon sequencing, copy number analysis, and RNA sequencing, have confirmed the high degree of heterogeneity exhibited by these tumors (9). These authors identified over 170 possible carcinogenic driver genes. These included well-documented oncogenes including NOTCH1, PHF6, FBXW7, USP7, PTHEN, DNM2, and BCL11B, as well as novel targets such as CCND3, MYB, CTCF, MED12, SMARCA4, CREBBP, and USP9X. These genes are closely related to a variety of dysfunctional signaling pathways including the Notch, JAK/STAT, PI3K/AKT/mTOR, and MAPK pathways.

Several genes were herein found to be overexpressed in T-ALL. These included LAT (linker for activation of T cells), and the LAT complex can facilitate TCR-mediated signaling that results in the activation of various downstream pathways that govern TCR-mediated activity (10). LAT is well established as a key mediator of signal transduction in T cells, with early studies of Jurkay T cells lacking LAT revealing an inability to promote Ca^2+^ mobilization, CD69 upregulation, ERK activation, Ras activation, and the transcription of AP-1/NFAT-regulated genes in response to TCR signaling (11). Reintroducing LAT in these cells can reverse these defects, underscoring its importance in the context of TCR signaling. Studies in mice have also shown that LAT is vital for T cell development, with the knockout of this gene resulting in impaired thymocyte development and an absence of mature αβ T cells in the periphery of these animals (12). Following TCR activation, phosphorylated LAT can serve as an adapter to which many other signaling proteins can bind (10). These proteins, in turn, can attract a range of chaperones in the cytosol and trigger additional tyrosine phosphorylation and protein-protein interaction. LAT is thus an important regulator of multi-protein signaling complex formation and associated regulatory activity, serving as an important nucleation site on the plasma membrane.

ITK (IL-2-induced T cell kinase) is another important mediator of TCR signaling that is known to be particularly important for Th2 responses and the induction of B cell-mediated humoral immunity (13). ITK signaling is also vital for the development of T cells (14), and mice lacking ITK expression or harboring a mutant isoform of this protein fail to effectively initiate Th2 responses under conditions that would typically engage a robust humoral immune response (15, 16). Moreover, the conditional knockout or mutation of ITK can contribute to increases in Th1-related cytokine production and T-bet expression (17). Elevated IL-4 levels observed in the context of the knockout of ITK have been suggested to be linked to weaker Th2 responses and Th1 cell expansion in a manner that may contribute to certain parasitic infections and allergic diseases (18). T cells that lack functional ITK expression are themselves dysfunctional but may serve as robust cytotoxic effectors with the potential to mediate antitumor responses (19). Studies of ITK signaling in the pathogenesis of diseases including malignant tumors and autoimmunity are thus an active area of ongoing clinical and preclinical research.

CD247, also referred to as CD3 ζ, CD3H, CD3Q, CD3Z, IMD25, T3Z, and TCRZ, is primarily expressed by T cells and natural killer (NK) cells. The CD3 ζ protein contains 6 tyrosine phosphorylation sites in its cytoplasmic domain that are phosphorylated in response to TCR activation (20). These phosphorylated tyrosine residues form an ITAM domain. While the ζ chain is not essential for the development of T cells, it is nonetheless required for TCR selection and preventing autoimmunity (21). Appropriate and sustained ζ chain phosphorylation is dependent on TCR ligand binding. When an αβ TCR recognizes a ligand that differs slightly from its cognate ligand, ζ phosphorylation is blocked in the middle stage such that downstream signal transduction is disrupted. Appropriate ζ phosphorylation can also protect against inappropriate T cell activation, with multi-stage phosphorylation being integral to the kinetics of T cell activation. TCR signaling can trigger a range of intracellular signaling events including inositol phospholipid hydrolysis, increased intracellular calcium levels, and MAPK signaling that ultimately promotes lymphokine upregulation and the proliferation and differentiation of these cells. TCR-CD3 ζ chain proteins are important for initiating signaling events following the activation of T cells. ITAM phosphorylation in the context of T cell activation enables the generation of signaling diversity in response to TCR activation (22).

The PRKCQ/PKC θ serine/threonine kinase is expressed at high levels by various hematopoietic cell types including T cells, NK cells, mast cells, and platelets, in addition to being expressed in the liver, nervous system, thymus, and skeletal muscle. PRKCQ is known to exhibit a range of immunological functions, with PRKCQ-deficient mice exhibiting impaired T cell activation as a consequence of the disruption of normal Ca^2+^ signal transduction activity and associated suppression of NF-kB and NFAT activation (23, 24). PRKCQ can further influence the expression of pro- and anti-apoptotic members of the Bcl-2 protein family to shape the survival of T cells, and it serves as an essential mediator of immune response to bacterial and viral pathogens (25). PRKCQ expression in certain gastrointestinal stromal tumors has similarly been shown to govern the ability of these cells to proliferate, and in breast epithelial cells it can enable survival, proliferation, and migration independent of growth factor signaling *via* the kinase-dependent activation of ERK/MAPK signaling (26). PRKCQ can also facilitate the growth of triple-negative breast cancer (TNBC) cells *in vitro* and *in vivo*, directly inhibiting ER expression in breast cancer cells. By stabilizing Fra-1 expression, PRKCQ can also stimulate the migration of TNBC cells while promoting the epithelial-mesenchymal transition in these breast tumor cells through the phosphorylation and activation of LSD1, thus supporting breast cancer growth and dissemination (27).

TRAT1 (TCR-related transmembrane junction 1) is a key TCR regulatory gene associated with tumor progression (28). IL-4 is capable of triggering cytotoxic responses in IL-17-producing CD8+ T cells (Tc17 cells), with IL-4/AKT signaling inducing TRAT1 upregulation and TCR stabilization in these Tc17 cells, thus enhancing their cytotoxic activity (29). TRAT1 expression is closely related to immune activity and the degree of infiltration by a range of immune cell populations including CD8+, cytotoxic, Th1, Th17, and Treg cells (30).

Roughly 20% of patients with T-ALL will develop recurrent disease, and prognostic outcomes in these individuals are poor. Genome-wide analyses of leukemia-associated genes in cases of recurrence have revealed that recurrent tumor cells generally express genes associated with resistance to cytotoxic chemotherapeutic treatment such as NT5C2 and MSH6 together with changes in the activation of many pathways such as the JAK/STAT and MAPK pathways. Accumulating genetic changes generally contribute to the incidence of recurrence after initiating treatment. Tzoneva et al., for example, found that NT5C2 mutations were present in 19% of individuals with T-ALL and related to resistance to treatment with nucleoside analogs such as mercaptopurine and thioguanine (31). Jones et al. additionally confirmed that MAPK pathway activity was altered at the time of recurrence (32), and they found these changes to be linked to corticosteroid resistance such that MAPK inhibitor treatment was sufficient to restore corticosteroid sensitivity in preclinical model systems. Inhibiting MAPK activity may thus be a means of treating ALL recurrence. In the present study, genes expressed in T-ALL cells were associated with TCR pathway signaling, adaptive immunity, intracellular signaling transduction, the plasma membrane, cell-cell junction, and protein kinase C activity, suggesting the importance of these pathways and compartments in this form of cancer. Raman spectra from T-ALL patients additionally confirmed that these pathways are significantly activated in T-ALL, supporting their potential value as diagnostic or prognostic biomarkers. These results also confirmed that collagen synthesis was significantly enhanced in T-ALL, accumulating in the extracellular space. Intercellular communication may thus represent an important area of future research focus in preclinical and clinical fields.

Advances in high-throughput sequencing technologies have led to a deeper understanding of the pathogenesis of AML and other myeloid tumor types. AML cases are characterized by the acquisition of multiple somatic mutations affecting various genes such that the disease evolves over time. In general, AML-related mutations tend to occur in an ordered manner, with mutations in epigenetic modifier-encoding genes including DNMT3A, ASXL1, TET2, IDH1, and IDH2 most often occurring early in the diseases. These mutations can persist even following treatment such that mutates cells can undergo clonal expansion while patients are in remission, eventually leading to recurrent disease. Mutations affecting NPM1 or signaling molecules including RAS and FLT3, in contrast, are generally secondary and arise during the more advanced stages of this disease (33). The resultant mutations and alterations in gene expression ultimately contribute to the differences in the functionality of AML cells relative to healthy hematopoietic stem progenitor cells. Large volumes of transcriptomic data can thus be leveraged to guide disease classification, risk stratification, and patient care.

Genes identified as being significantly overexpressed in AML in this study included TLR4, MPO, MNDA, CSF1R, and C3AR1. The pattern recognition receptor TLR4 (Toll-like receptor 4) is capable of binding to endogenous or exogenous ligands, and it is expressed by AML cells as well as a range of cells in the bone marrow stroma and inflammation-regulating cell subsets. Various endogenous ligands can bind TLR4 within the bone marrow microenvironment, and it is also expressed on the surface of non-leukemic bone marrow cells including mesenchymal cells, endothelial cells, differentiated bone marrow cells, and inflammatory or immunologically active cells. Osteoblasts serve as important supporting cells found within the stem cell niche that can enable the ongoing survival and proliferation of primary AML cells. This support is facilitated by bilateral cytokine-mediated crosstalk between osteoblasts and AML cells. TLR4 is involved in defense mechanisms in neutropenic patients suffering from infections, and it can regulate immune response induction and inflammation in individuals undergoing allogeneic stem cell transplantation. TLR4 can thus directly impact primary AML cells, in addition to indirectly influencing these cells through the regulation of proximal cells in the bone marrow stroma. TLR4 is also capable of regulating inflammatory activity and anti-leukemic immune responses in allograft recipients.

Myeloperoxidase (MPO) is a heme-containing peroxidase protein that is expressed at high levels in a range of immune cell types such as neutrophils, monocytes/macrophages, and activated microglia, in addition to being present in neurons and astrocytes (34). Genetic variations in MPO are associated with a higher risk of ischemic stroke (35). The activation and degranulation of neutrophils is also particularly important for MPO induction, serving as the primary source of MPO found in the plasma. Neutrophil-derived MPO activity reaches peak levels on days 1-3 after a stroke, while macrophage/microglia-derived MPO activity peaks on days 5-7. An anti-neutrophil monoclonal antibody (RP3) is capable of inhibiting the activity of MPO within 24 h of injection, thereby decreasing brain edema and cerebral infarction in ischemic brain tissue. The extent of neutrophil-mediated MPO activity is thus a key determinant of ischemic stroke-related inflammatory activity and brain damage.

MNDA (myeloid nuclear differentiation antigen) is expressed at high levels in granulocytes, monocytes, and certain subsets of B cells including those lymphocytes present in marginal regions of the spleen (36, 37). Mechanistically, it serves to regulate type I interferon signaling in cells of the bone marrow. MNDA, unlike IFI16, is not a sensor for dsDNA, but it is required for the induction of type I IFN responses through its ability to induce IRF7. MNDA is also required for appropriate enhancer formation on the IRF7 promoter in humans, being recruited to this gene promoter in response to signaling through the interferon receptor. MNDA thus serves as a vital regulator of type I interferon signaling activity in human bone marrow cells (36).

CSF1R (colony-stimulating factor 1 receptor) serves as an essential regulator of the survival and differentiation of macrophages and other mononuclear phagocytes. As a type III protein tyrosine kinase receptor, CSF1R can homodimerize upon interaction with IL-34 or CSF1, thereby triggering downstream signaling activity (38). The presence of macrophages expressing CSF1R within tumors is linked to low survival rates in a variety of cancers, indicating that efforts to target these CSF1R+ tumor-associated macrophages may aid in tumor clearance. CSF1R is also expressed by other tumor-associated myeloid cells including neutrophils, dendritic cells, and myeloid-derived suppressor cells (MDSCs). Holgaard et al. demonstrated that following treatment with the CSF1R small molecule inhibitor PLX3397, MDSCs underwent reprogramming and differentiation to yield pro-inflammatory cells capable of killing tumor cells (39). Owing to phenotypic heterogeneity, further research is needed to clarify the functions of MDSCs in the context of inflammation in humans and mice. Additional studies of CSF1/CSF1R-mediated signal transduction in non-macrophage human myeloid cells are warranted.

The transmembrane G-protein-coupled receptor C3aR1 serves as a receptor for the activated C3a complement protein (40). C3aR1 expression has been observed in the pancreatic, brain, and adipose tissue, and its expression is increased in obese rodents consuming high-fat diets (41). C3aR1 expression is primarily observed in immune cells including macrophages, but it is also expressed by adipocytes and a range of other stromal cell types. When activated by C3a binding, protein activation and β Arrestin1/2 recruitment trigger the downstream activation of diverse signaling pathways, leading to a net increase in intracellular calcium ion levels (41). At present, 3D structural information pertaining to C3aR1 is not available. The sequence homology between human C3aR1 and C5aR1 is ~57%, and X-ray crystallography has been successfully applied to clarify the structure of C5aR1, offering a tool for use in C3aR1 homology modeling (42).

In this study, we analyzed the differential expression of several important genes identified in previous studies, finding that there were indeed significant differences in the expression of these genes in AML and T-ALL. The results of the bioinformatics analysis were verified using Raman spectroscopy. The results indicated that the results of the analyses were both detailed and reliable. Furthermore, our findings were consistent with those of other research and experimental results. Svojgr et al. analyzed the mRNA levels and cell surface expression of LAT using RT-PCR and flow cytometry, and the results showed significant increases in LAT with the maturation of both malignant and non-malignant precursors in T-ALL cases (43). Dombroski et al. demonstrated that ITK has signaling potential independent of its kinase activity, while Wenchang Guo et al. reported that inhibiting ITK can reduce the proliferation and growth of malignant T cell tumor cells (44, 45). Although there are no reports showing significant associations between CD247 and the occurrence and progression of T-ALL, TCR-CD3 ζ chains have been shown to play a crucial role in the initiation of proximal signaling events after T cell activation, which may provide new directions for future research. Schmitt et al. analyzed differences in the mRNA and protein expression of TLR-4 in DC cells from AML patients and normal donors using qRT PCR, Western blotting, and flow cytometry (46). The results showed that both AML and normal cells expressed TLR-4 at high levels without no significant difference between them. These findings are consistent with our experimental results, namely, that TLR-4 is significantly overexpressed in the cells of AML patients. According to the classification of the World Health Organization (WHO, 2008), AML can be evaluated and differentiated from ALL using flow cytometry (FCM)/immunohistochemistry (IHC)/cytochemistry techniques, with myeloperoxidase (MPO) being one of the clear markers for differentiation (47). The various data in the above literature further verify that the key genes identified in the present study are consistent with our bioinformatics analysis and experimental verification results in terms of their protein expression.

The present Raman spectroscopy results revealed that the hematopoietic microenvironment associated with T-ALL incidence was significantly impacted by excessive leukemic cell proliferation, resulting in the altered degradation and synthesis of collagen in the extracellular matrix. As T-ALL patients may face an elevated risk of liver damage and consequent increases in levels of serum ADA, the nucleic acid levels in their bone marrow samples were reduced. This may be related to the expression of important genes including FOS, TLR4, and CD44. Increased metabolic activity in the bone marrow of T-ALL patients may also contribute to higher levels of amino acid and protein utilization such that the peripheral blood protein levels in these patients were decreased as compared to patients with AML, potentially indicating that these patients may face a poorer prognosis. Owing to the limited number of samples available for this study, the analyzed biochemical parameters did not differ significantly when comparing patient peripheral blood samples. In contrast, the Raman spectroscopy results were able to highlight differences between these two groups of cancer patients, emphasizing the enhanced sensitivity of Raman spectra-based analyses of bone marrow supernatant samples and their ability to reflect relatively subtle differences not evident in peripheral blood samples, thus enabling the more reliable identification of biomarkers of interest. Other factors such as patient disease history, drug history, smoking and drinking history were not analyzed in depth in this study yet may have influenced the resultant data. Accordingly, future large-scale analysis with standardized approaches to data collection will be necessary to achieve enhanced sampling accuracy.

ADA is an adenosine deaminase that plays a unique and important role in the differentiation and maturation of the immune system. The activity of ADA in the thymus is much higher than that in other organs. In lymphocytes, the ADA activity in cortical thymocytes is higher than that in medullary thymocytes and peripheral lymphocytes, while its activity in T cells is higher than that in B cells (48, 49). Studies have found that the immunoreactive ADA protein and translatable ADA mRNA in T lymphoblastic cell lines are 6-8 times higher than those in B lymphoblastic cell lines, which corresponds to the higher ADA catalytic activity and protein ratio observed in T cells compared to B cells (50). These observations indicate that there may be fundamental differences in the degradation rate of ADA protein and nucleotide metabolism between T cells and B cells, as well as among members of the thymic cell lineage at different stages of maturation. In lymphocytes, cell surface ADA not only degrades extracellular adenosine, but also regulates the action of adenosine mediated by A2B subtype adenosine receptors (51). Our research results showed that the expression of ADA protein in T-ALL tumor cells was higher than that in tumor cells from AML patients, suggesting a specific difference in ADA-mediated signal transduction and cytokine secretion between the two types of leukemia. Human serum albumin (HSA, ALB) is the most abundant protein in plasma, and is the main determinant of plasma swelling pressure and the main regulator of fluid distribution between various parts of the body (52). ALB is the main carrier of fatty acids (FA), and can affect the pharmacokinetics of many drugs and metabolically modify several ligands, as well as rendering potential toxins harmless. It is a source of the plasma antioxidant capacity (53). HSA is a biomarker for many diseases, including cancer, rheumatoid arthritis, local ischemia, postmenopausal obesity, severe acute graft versus host disease, and diseases that require the monitoring and control of blood glucose (54). Therefore, the concentration of albumin is a very important clinical indicator. From our test results, it can be seen that both T-ALL and AML patients have elevated albumin expression in their cells, indicating that many biological reactions requiring albumin such as inflammation have occurred in the patient’s body, although the difference between the levels in T-ALL and AML patients was not found to be significant.

AML is a highly heterogeneous disease such that even with ongoing advances in the clinical treatment of this disease, most AML patients still face a poor prognosis. AML-focused research to date has largely centered around the characterization of signaling pathways and metabolic processes that may yield a foundation for novel forms of anti-leukemia treatment in the future. The design of novel therapeutic agents targeting the apoptosis, RTK signaling, HH signaling, DDR, transcriptional regulation, and mitochondrial metabolism pathways has the potential to improve patient outcomes. LSCs are also thought to be major drivers of the progression and persistence of this devastating disease. In the present study, AML cells were found to express a range of genes associated with inflammatory activity, immune response induction, antigen presentation, MHC class II activation, and cytokine production, potentially offering a foundation for the clinical efforts to treat affected patients. Raman spectroscopy was employed to validate the present results based on an analysis of primary AML patient-derived cells. These results confirmed that relative to T-ALL patients, patients with AML exhibited higher levels of nucleic acid and protein synthesis pathway activity related to the high levels of immune-related activity, inflammation, mitochondrial respiration, and cytokine-associated activities engaged by tumor cells in AML patients. Overall, these data demonstrated that AML-related gene expression profiles are complex and dynamic such that combination treatment strategies may ultimately yield the greatest degree of therapeutic efficacy.

## Conclusions

Advances in molecular research have helped enable the design of small molecule drugs targeting a range of pathways for the treatment of AML and T-ALL, yet the prognosis of patients with these hematological malignancies remains relatively poor. In the present study, a bioinformatics approach was used to compare the transcriptomic profiles of AML and T-ALL patients, ultimately revealing clear differences in signal transduction pathway activity and PPI networks for these two cancer types. Raman spectra were further used to validate these bioinformatics results, confirming important regulatory roles for genes including LAT, ITK, CD247, TLR4, and MPO when differentiating between these two diseases, revealing a higher demand for nucleic acid and protein synthesis in AML patient cells. As such, efforts to target these genes and pathways may offer a basis for the development of novel pharmacological interventions aimed at more effectively treating affected patients. While future in-depth experimental analyses will be essential to validate these results, they nonetheless provide a promising pathway toward the improvement of prognostic outcomes in leukemia patients.

## Data availability statement

The datasets presented in this study can be found in online repositories. The names of the repository/repositories and accession number(s) can be found within the article/**Supplementary Materials**.

## Ethics statement

The studies involving human participants were reviewed and approved by the Ethics Committee of the Blood Diseases Hospital of the Chinese Academy of Medical Sciences. Written informed consent to participate in this study was provided by the participants’ legal guardian/next of kin.

## Author contributions

HL, XK and ZC contributed equally to this work. YZ, QZ and JQ conceived and directed the project. HL performed the experiments. HL, XK, ZC, HW, EL and FS analyzed the data. HL wrote the manuscript. XK, ZC, HW, EL, FS, JQ, QZ and YZ contributed to the discussions and comments on the paper. All authors contributed to the article and approved the submitted version.

## References

[1] Wei W Yang D Chen X Liang D Zou L Zhao X. Chimeric antigen receptor T-cell therapy for T-ALL and AML. Front Oncol (2022) 12:967754. doi: 10.3389/fonc.2022.967754 36523990 PMC9745195

[2] Carter JL Hege K Yang J Kalpage HA Su Y Edwards H. Targeting multiple signaling pathways: the new approach to acute myeloid leukemia therapy. Signal Transduct Target Ther (2020) 5:288. doi: 10.1038/s41392-020-00361-x 33335095 PMC7746731

[3] Patel AA Thomas J Rojek AE Stock W. Biology and treatment paradigms in T cell acute lymphoblastic leukemia in older adolescents and adults. Curr Treat Options Oncol (2020) 21:57. doi: 10.1007/s11864-020-00757-5 32468488

[4] Raetz EA Teachey DT. T-Cell acute lymphoblastic leukemia. hematology. Am Soc Hematol (2016) 2016:580–8. doi: 10.1182/asheducation-2016.1.580 PMC6142501 27913532

[5] Durinck K Goossens S Peirs S Wallaert A Van Loocke W Matthijssens F. Novel biological insights in T-cell acute lymphoblastic leukemia. Exp Hematol (2015) 2015)43:625–39. doi: 10.1016/j.exphem.2015.05.017 26123366

[6] Patrick K Vora A. Update on biology and treatment of T-cell acute lymphoblastic leukaemia. Curr Opin Pediatr (2015) 27:44–9. doi: 10.1097/MOP.0000000000000171 25502893

[7] Van Vlierberghe P Ferrando A. The molecular basis of T cell acute lymphoblastic leukemia. J Clin Invest (2012) 122:3398–406. doi: 10.1172/JCI61269 PMC3461904 23023710

[8] Kraszewska MD Dawidowska M Szczepański T Witt M. T-Cell acute lymphoblastic leukaemia: recent molecular biology findings. Br J Haematol (2012) 156:303–15. doi: 10.1111/j.1365-2141.2011.08957.x 22145858

[9] Liu Y Easton J Shao Y Maciaszek J Wang Z Wilkinson MR. The genomic landscape of pediatric and young adult T-lineage acute lymphoblastic leukemia. Nat Genet (2017) 49:1211 – 8. doi: 10.1038/ng.3909 PMC5535770 28671688

[10] Balagopalan L Coussens NP Sherman E Samelson LE Sommers CL. The LAT story: a tale of cooperativity, coordination, and choreography. Cold Spring Harbor Perspect Biol (2010) 2:a005512. doi: 10.1101/cshperspect.a005512 PMC2908767 20610546

[11] Finco TS Kadlecek T Zhang W Samelson L Weiss A. LAT is required for TCR-mediated activation of PLCgamma1 and the ras pathway. Immunity (1998) 9:617–26. doi: 10.1016/S1074-7613(00)80659-7 9846483

[12] Fruman DA Rameh LE Cantley LC. Phosphoinositide binding domains: embracing 3-phosphate. Cell (1999) 97:817–20. doi: 10.1016/S0092-8674(00)80792-8 10399908

[13] Andreotti AH Schwartzberg PL Joseph RE Berg LJ. T-Cell signaling regulated by the tec family kinase, itk. Cold Spring Harbor Perspect Biol (2010) 2:a002287. doi: 10.1101/cshperspect.a002287 PMC2890196 20519342

[14] Liao XC Littman DR. Altered T cell receptor signaling and disrupted T cell development in mice lacking itk. Immunity (1995) 3:757–69. doi: 10.1016/1074-7613(95)90065-9 8777721

[15] Fowell DJ Shinkai K Liao XC Beebe AM Coffman RL Littman DR. Impaired NFATc translocation and failure of Th2 development in itk-deficient CD4+ T cells. Immunity (1999) 11:399–409. doi: 10.1016/S1074-7613(00)80115-6 10549622

[16] Schaeffer EM Yap GS Lewis CM Czar MJ McVicar DW Cheever AW. Mutation of tec family kinases alters T helper cell differentiation. Nat Immunol (2001) 2:1183–8. doi: 10.1038/ni734 11702066

[17] Miller AT Wilcox HM Lai Z Berg LJ. Signaling through itk promotes T helper 2 differentiation *via* negative regulation of T-bet. Immunity (2004) 21:67–80. doi: 10.1016/j.immuni.2004.06.009 15345221

[18] Kannan AK Mohinta S Huang W Huang L Koylass N Appleton JA. T-Bet independent development of IFNγ secreting natural T helper 1 cell population in the absence of itk. Sci Rep (2017) 7:45935. doi: 10.1038/srep45935 28406139 PMC5390256

[19] Hewitt SL Bailey D Zielinski J Apte A Musenge F Karp R. Intratumoral IL12 mRNA therapy promotes TH1 transformation of the tumor microenvironment. Clin Cancer Res (2020) 26:6284–98. doi: 10.1158/1078-0432.CCR-20-0472 32817076

[20] Tao WA Wollscheid B O'Brien R Eng JK Li XJ Bodenmiller B. Quantitative phosphoproteome analysis using a dendrimer conjugation chemistry and tandem mass spectrometry. Nat Methods (2005) 2:591–8. doi: 10.1038/nmeth776 16094384

[21] Labadia ME Ingraham RH Schembri-King J Morelock MM Jakes S. Binding affinities of the SH2 domains of ZAP-70, p56lck and shc to the zeta chain ITAMs of the T-cell receptor determined by surface plasmon resonance. J Leuk Biol (1996) 59:740–6. doi: 10.1002/jlb.59.5.740 8656061

[22] Osman N Turner H Lucas S Reif K Cantrell DA. The protein interactions of the immunoglobulin receptor family tyrosine-based activation motifs present in the T cell receptor zeta subunits and the CD3 gamma, delta and epsilon chains. Eur J Immunol (1996) 26:1063–8. doi: 10.1002/eji.1830260516 8647168

[23] Pfeifhofer C Kofler K Gruber T Tabrizi NG Lutz C Maly K. Protein kinase c theta affects Ca2+ mobilization and NFAT cell activation in primary mouse T cells. J Exp Med (2003) 197:1525–35. doi: 10.1084/jem.20020234 PMC2193906 12782715

[24] Altman A Kaminski S Busuttil V Droin N Hu J Tadevosyan Y. Positive feedback regulation of PLCgamma1/Ca(2+) signaling by PKCtheta in restimulated T cells *via* a tec kinase-dependent pathway. Eur J Immunol (2004) 34:2001–11. doi: 10.1002/eji.200324625 15214048

[25] Valenzuela JO Iclozan C Hossain MS Prlic M Hopewell E Bronk CC. PKCtheta is required for alloreactivity and GVHD but not for immune responses toward leukemia and infection in mice. J Clin Invest (2009) 119:3774–86. doi: 10.1172/JCI39692 PMC2786796 19907075

[26] Byerly J Halstead-Nussloch G Ito K Katsyv I Irie HY. PRKCQ promotes oncogenic growth and anoikis resistance of a subset of triple-negative breast cancer cells. Breast Cancer Res (2016) 18:95. doi: 10.1186/s13058-016-0749-6 27663795 PMC5034539

[27] Boulding T McCuaig RD Tan A Hardy K Wu F Dunn J. LSD1 activation promotes inducible EMT programs and modulates the tumour microenvironment in breast cancer. Sci Rep (2018) 8:73. doi: 10.1038/s41598-017-17913-x 29311580 PMC5758711

[28] Quan Q Xiong X Wu S Yu M. Identification of immune-related key genes in ovarian cancer based on WGCNA. Front Genet (2021) 12:760225. doi: 10.3389/fgene.2021.760225 34868239 PMC8634599

[29] Liu CH Lin BS Wu MY Song YC Ke TW Chou YL. Adoptive transfer of IL-4 reprogrammed Tc17 cells elicits anti-tumour immunity through functional plasticity. Immunology (2022) 166:310–26. doi: 10.1111/imm.13473 PMC11558351 35322421

[30] Xiao XY Guo Q Tong S Wu C Chen J Ding Y. TRAT1 overexpression delays cancer progression and is associated with immune infiltration in lung adenocarcinoma. Front Oncol (2022) 12:960866. doi: 10.3389/fonc.2022.960866 36276113 PMC9582843

[31] Zhang J Ding L Holmfeldt L Wu G Heatley SL Payne-Turner D. The genetic basis of early T-cell precursor acute lymphoblastic leukaemia. Nature (2012) 481:157–63. doi: 10.1038/nature10725 PMC3267575 22237106

[32] Jones CL Gearheart CM Fosmire S Delgado-Martin C Evensen NA Bride K. MAPK signaling cascades mediate distinct glucocorticoid resistance mechanisms in pediatric leukemia. Blood (2015) 126:2202–12. doi: 10.1182/blood-2015-04-639138 PMC4635116 26324703

[33] Bullinger L Döhner K Döhner H. Genomics of acute myeloid leukemia diagnosis and pathways. J Clin Oncol (2017) 35:934–46. doi: 10.1200/JCO.2016.71.2208 28297624

[34] Yu G Liang Y Huang Z Jones DW Pritchard KA Jr Zhang H. Erratum to: inhibition of myeloperoxidase oxidant production by n-acetyl lysyltyrosylcysteine amide reduces brain damage in a murine model of stroke. J Neuroinflam (2016) 13:166. doi: 10.1186/s12974-016-0639-y PMC4924260 27349966

[35] Manso H Krug T Sobral J Albergaria I Gaspar G Ferro JM. Variants in the inflammatory IL6 and MPO genes modulate stroke susceptibility through main effects and gene-gene interactions. J Cereb Blood Flow Metab (2011) 31:1751–9. doi: 10.1038/jcbfm.2011.27 PMC3170942 21407237

[36] Righi S Novero D Godio L Bertuzzi C Bacci F Agostinelli C. Myeloid nuclear differentiation antigen: an aid in differentiating lymphoplasmacytic lymphoma and splenic marginal zone lymphoma in bone marrow biopsies at presentation. Hum Pathol (2022) 124:67–75. doi: 10.1016/j.humpath.2022.03.008 35339566

[37] Fotouhi-Ardakani N Kebir DE Pierre-Charles N Wang L Ahern SP Filep JG. Role for myeloid nuclear differentiation antigen in the regulation of neutrophil apoptosis during sepsis. Am J Respir Crit Care Med (2010) 182:341–50. doi: 10.1164/rccm.201001-0075OC 20395555

[38] Achkova D Maher J. Role of the colony-stimulating factor (CSF)/CSF-1 receptor axis in cancer. Biochem Soc Trans (2016) 44:333–41. doi: 10.1042/BST20150245 27068937

[39] Holmgaard RB Zamarin D Lesokhin A Merghoub T Wolchok JD. Targeting myeloid-derived suppressor cells with colony stimulating factor-1 receptor blockade can reverse immune resistance to immunotherapy in indoleamine 2,3-dioxygenase-expressing tumors. EBioMedicine (2016) 6:50–8. doi: 10.1016/j.ebiom.2016.02.024 PMC4856741 27211548

[40] Hollmann TJ Haviland DL Kildsgaard J Watts K Wetsel RA. Cloning, expression, sequence determination, and chromosome localization of the mouse complement C3a anaphylatoxin receptor gene. Mol Immunol (1998) 35:137–48. doi: 10.1016/S0161-5890(98)00021-2 9694514

[41] Sahu BS Rodriguez P Nguyen ME Han R Cero C Razzoli M. Peptide/Receptor Co-evolution explains the lipolytic function of the neuropeptide TLQP-21. Cell Rep (2019) 28:2567–80. doi: 10.1016/j.celrep.2019.07.101 PMC6753381 31484069

[42] Robertson N Rappas M Doré AS Brown J Bottegoni G Koglin M. Structure of the complement C5a receptor bound to the extra-helical antagonist NDT9513727. Nature (2018) 553:111–4. doi: 10.1038/nature25025 29300009

[43] Svojgr K Burjanivova T Vaskova M Kalina T Stary J Trka J. Adaptor molecules expression in normal lymphopoiesis and in childhood leukemia. Immunol Lett (2009) 122:185–92. doi: 10.1016/j.imlet.2008.12.008 19183565

[44] Dombroski D Houghtling RA Labno CM Precht P Takesono A Caplen NJ. Kinase-independent functions for itk in TCR-induced regulation of vav and the actin cytoskeleton. J Immunol (Baltimore Md 1950) (2005) 174:1385–92. doi: 10.4049/jimmunol.174.3.1385 15661896

[45] Guo W Liu R Ono Y Ma AH Martinez A Sanchez E. Molecular characteristics of CTA056, a novel interleukin-2-inducible T-cell kinase inhibitor that selectively targets malignant T cells and modulates oncomirs. Mol Pharmacol (2012) 82:938–47. doi: 10.1124/mol.112.079889 PMC3477223 22899868

[46] Schmitt A Li L Giannopoulos K Greiner J Reinhardt P Wiesneth M. Quantitative expression of toll-like receptor-2, -4, and -9 in dendritic cells generated from blasts of patients with acute myeloid leukemia. Transfusion (2008) 48:861–70. doi: 10.1111/j.1537-2995.2007.01616.x 18208411

[47] Ahuja A Tyagi S Seth T Pati HP Gahlot G Tripathi P. Comparison of immunohistochemistry, cytochemistry, and flow cytometry in AML for myeloperoxidase detection. IJHBT (2018) 34:233–9. doi: 10.1007/s12288-017-0849-1 PMC5884971 29622864

[48] Chechik BE Schrader WP Minowada J. An immunomorphologic study of adenosine deaminase distribution in human thymus tissue, normal lymphocytes, and hematopoietic cell lines. J Immunol (Baltimore Md 1950) (1981) 126:1003–7. doi: 10.4049/jimmunol.126.3.1003 7007497

[49] Sullivan JL Osborne WR Wedgewood RJ. Adenosine deaminase activity in lymphocytes. BRIT J HAEMATOL (1977) 37:157–8. doi: 10.1111/j.1365-2141.1977.tb08825.x 412512

[50] Wiginton DA Adrian GS Friedman RL Suttle DP Hutton JJ. Cloning of cDNA sequences of human adenosine deaminase. P Natl Acad Sci USA (1983) 80:7481–5. doi: 10.1073/pnas.80.24.7481 PMC389975 6200875

[51] Herrera C Casadó V Ciruela F Schofield P Mallol J Lluis C. Adenosine A2B receptors behave as an alternative anchoring protein for cell surface adenosine deaminase in lymphocytes and cultured cells. Mol Pharmacol (2001) 59:127–34. doi: 10.1124/mol.59.1.127 11125033

[52] Mendez CM McClain CJ Marsano LS. Albumin therapy in clinical practice. Nutr Clin Pract Off Publ Am Soc Parenteral Enteral Nutr (2005) 20:314–20. doi: 10.1177/0115426505020003314 16207669

[53] Ascenzi P Fasano M. Allostery in a monomeric protein: the case of human serum albumin. Biophys Chem (2010) 148:16–22. doi: 10.1016/j.bpc.2010.03.001 20346571

[54] Sbarouni E Georgiadou P Voudris V. Ischemia modified albumin changes - review and clinical implications. Clin Chem Lab Med (2011) 49:177–84. doi: 10.1515/CCLM.2011.037 21083441

