# Supplementary material for: Bioinformatics and Raman spectroscopy-based identification of key pathways and genes enabling differentiation between acute myeloid leukemia and T cell acute lymphoblastic leukemia
Source: Front Immunol. 2023 May 17;14:1194353. doi: 10.3389/fimmu.2023.1194353 (PMC10229868; doi:10.3389/fimmu.2023.1194353)
Supplement: Supplementary file 1 [file DataSheet_1.pdf]

Supplementary Material

Bioinformatics and Raman Spectroscopy-based Identification of Key Pathways and Genes Enabling Differentiation Between Acute Myeloid Leukemia and T Cell Acute Lymphoblastic Leukemia

Haoyue Liang[1,2#], Xiaodong Kong[3#], Zhijie Cao[1,2#], Haoyu Wang[1,2], Ertao Liu[1,2], Fanfan Sun[1,2], Jianwei Qi[1,2*], Qiang Zhang[3*], Yuan Zhou[1,2*]

*** Correspondence:**

Yuan Zhou
yuanzhou@ihcams.ac.cn

Qiang Zhang
zhangqiangyulv@163.com

Jianwei Qi
qijianwei@ihcams.ac.cn

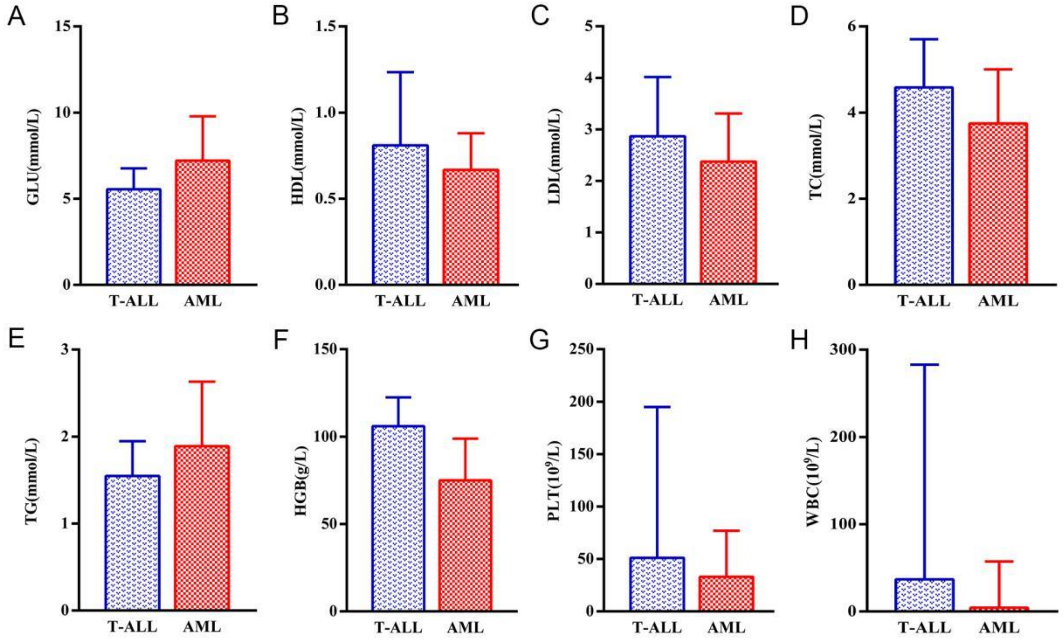

Supplementary Figure 1 Serum biochemistry results in A. Glucose; B. High-density lipoprotein; C. Low-density lipoprotein; D. Total cholesterol; E. Triglycerides; F. Hemoglobin; G. Platelet; H. White blood cell

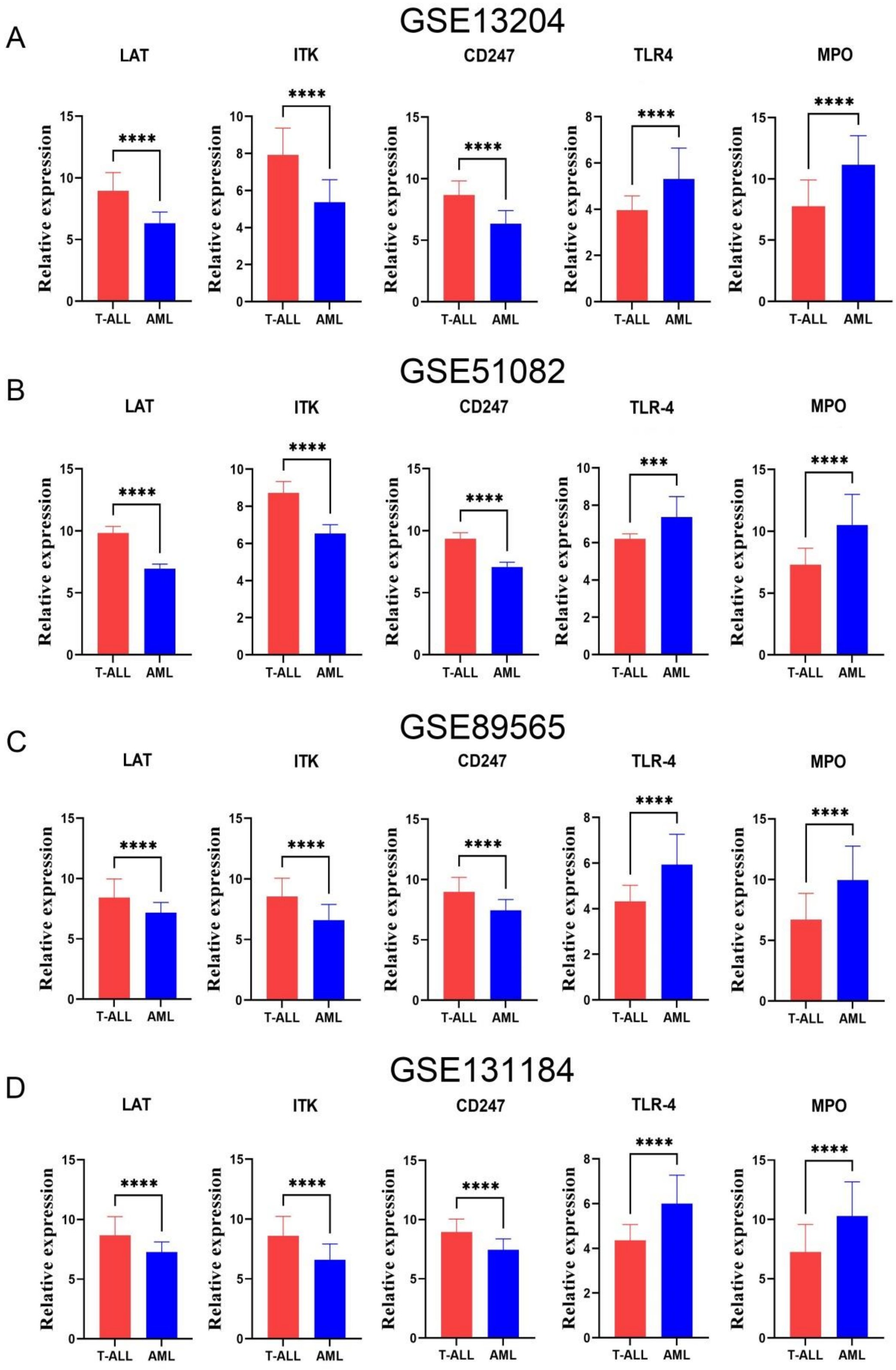

Supplementary Figure 2 Relative expression of LAT, ITK, CD247, TLR-4 and MPO A. GSE13024; B. GSE51082; C. GSE89565; D. GSE131184. ****P<0.0001.

Supplementary Table 1. Peak assignments for Raman spectra of bone marrow supernatant

Biochemical group	Peak position (cm^{-1})	Assignments	Reference
Proteins and amino acids	643	Tyr	(1, 3, 5)
	759	Trp	(1, 3, 5)
	1003	Phe	(1-3, 6)
	1260	Amide III	(1, 3, 4)
	1446	CH_2 bending mode	(1-3, 6)
	1548	Trp	(1)
	1603	Phe, Tyr	(1, 3, 4, 6)
	1616	Tyr, Trp	(1, 3, 6)
	1654	Amide I	(1, 3, 6)
Lipids	1446	Lipids	(2, 3, 6)
Nucleic acids	826	O–P–O stretch	(1, 2)
	1573	Adenine, guanine	(1)
	1579	Pyrimidine ring	(1, 6)
Collagen	859	Collagen type I	(1, 5)

Note: Abbreviations: Phe: phenylalanine; Tyr: tyrosine; Trp: tryptophan.

Supplementary Table 2. Comparison of clinical data from subjects assigned to the T-ALL and AML groups

	T-ALL	AML	Statistical value	*P* value
Gender Male(n)	1(3)	7(7)	χ^2=5.833	0.067
Age (years)	18.00±5.51	34.71±9.15	*t*=-1.127	0.292
WBC(*10^9/L)	36.73(5.27-282.98)	4.35(1.70-65.38)	Z=-1.254	0.210
PLT(*10^9/L)	51.00(44.00-195.00)	33.00(27.00-344.00)	Z=-0.798	0.425
HGB(g/L)	106.00±9.54	75.00±9.01	*t* =2.021	0.780
TP(g/L)	64.13±0.30	68.16±1.83	*t* =-1.385	0.203
GLU(mmol/L)	5.56±0.70	7.22±0.97	*t* =-1.047	0.326
TG(mmol/L)	1.55±0.23	1.89±0.28	*t* =-0.739	0.481
TC(mmol/L)	4.58±0.65	3.75±0.48	*t* =0.989	0.352
HDL(mmol/L)	0.81±0.25	0.67±0.08	*t* =0.736	0.483
LDL(mmol/L)	2.87±0.66	2.38±0.35	*t* =0.717	0.494
ADA(U/L)	171.53±139.54	26.39±3.27	*t* =1.040	0.407

Note: Data not conforming to the normal distribution are expressed using M (range).

Supplementary Table 3. Common DEG identified in GSE13204, GSE51082, GSE89565 and GSE131184

Gene symbol (up-regulated DEGs)
LPAR6, SH3BP5, PRKCH, PPP1R16B, RCBTB2, CR2, USP9Y, ITM2A, KDM5D, ZEB1, ABLIM1, SERINC5, TXLNGY, SCN3A, FXYD2, TRIB2, NINL, NMT2, LDLRAD4, IL32, ENO2, PRKCQ, ARMCX2, CLGN, TSHR, AEBP1, GPRASP1, IL7R, CD247, ARPP21, ITK, LAT, MLLT11, PVRIG, ZNF711, FAT1, RASGRP1, CD1B, ELOVL4, TRAT1, DNTT, TSPAN7, MAL

Supplementary Table 4. Common DEG identified in GSE13204, GSE51082, GSE89565 and GSE131184

Gene symbol (down-regulated DEGs)
CFD, AZU1, MPO, TUBB6, CPA3, PDLIM1, CYTL1, KIT, FLT3, CCNA1, HLA-DRA, CSTA, ELANE, F13A1, HLA-DPA1, PRTN3, ECRP, RNASE3, PPBP, CSF2RB, MS4A3, MNDA, LYN, CEBPD, CTSA, PDGFC, CA2, NRGN, HHEX, IGFBP7, CTSG, STAR, LYZ, KLF4, ADRB2, LGALS1, LY86, GALNT11, THEMIS2, ARL4A, MLC1, HOMER3, ACSL1, FCER1G, CST7, MEST, PTGS2, ZNF185, PLTP, PLAGL1, TENT5A, PROM1, CLC, CYTIP, SERPING1, IRS2, CTSB, FNDC3B, RAB13, TIMP1, HEBP1, STX11, NCF2, GSAP, MAN1A1, NFE2, FOS, HLA-DMB, CYFIP1, MAP3K5, BLVRB, SAMHD1, MS4A6A, CD36, PRKAR2B, ASL, ERLIN1, BST1, GCA, FGL2, RAB31, DUSP6, GNS, HLA-DPB1, MEF2C, TSPO, RASSF2, C3AR1, GLIPR1, PRG2, VCAN, PNP, CST3, HLA-DQB1, TST, TFEC, FUT4, CTSS, RNASE6, EGFL7, PSTPIP2, PTGER2, CSF1R, B4GALT5, CD44, S100A4, LILRA2, DYNLT1, TNFRSF1B, TLR4, S100A11, IGF2BP2, STOM, SKAP2, CD14, CCR1, CPD, RGS2, S100P, CD74, SELENOP, TNS3, LGALS2, FCN1, MSRB1, FCER1A, LXN, TRIB1, C5AR1

Supplementary Table 5. DEGs of AML vs T-ALL identified in GSE13204

Gene symbol (up-regulated DEGs)
MAL,LCK,CD3D,CHI3L2,CD3G,TSPAN7,CTHRC1,DNTT,TRAT1,TRBC1,ELOVL4,HHIP-AS1,CD1B,ZAP70,RASGRP1,GXYLT2,GATA3,SHISA2,BCL11B,FAT1,SH2D1A,CAMK4,PRTFDC1,LEF1,CD3E,ZNF711,MZB1,TCF7,SNTG2-AS1,CD2,UBASH3A,PVRIG,FAM110C,MLLT11,LAT,SEPTIN1,CD1E,CD7,ITK,TOX2,ZNF423,PCAT18,FZD6,TOX,RHOH,ARPP21,HHIP,CD247,CD1A,RTL6,PKIA,EVL,IL7R,LINC01215,ARL4C,CCR9,GPRASP1,AEBP1,FAM169A,SBK1,SCAI,KCNA3,IRX5,WAKMAR2,TSHR,CLGN,CRNDE,ARMCX2,CYFIP2,PRKCQ,PABPC4L,ETS1,ENO2,ADA,ZNF827,PARD3,RUFY3,CD8A,KIF3A,MYH10,NDST3,IL32,LINC01260,SLC7A3,PBK,PLCB4,LEF1-AS1,HES4,EPHB6,LOC100505501,SALL2,GIMAP6,LDLRAD4,PIK3C2B,NMT2,NINL,TRIB2,FXYD2,C12orf75,CDCA7,SCN3A,OCIAD2,FRMD6,MYEF2,MME,GIMAP7,RUBCNL,SLC23A1,MIR646HG,DBN1,SCGB3A1,P2RX5,LIG4,TMEM263,SELENOW,TXLNGY,ZNF57,H2BW4P,GALNT6,NLRC3,PXYLP1,OGN,TASP1,SLC37A3,TBC1D4,SCN2A,UHRF1,ZEB1-AS1,LBH,CRY1,TMEM131L,DBH-AS1,IKZF2,SERINC5,ID3,VPREB1,DENND2D,RAB15,SPRY1,SLC5A3,TRAC,IGF2R,THEMIS,LRP12,SLAIN1,IFITM1,LOC285097,STARD4,ABLIM1,SIT1,TFDP2,LUNAR1,ICOS,POU2AF1,TCFL5,ZEB1,LIME1,NID2,CHRNA3,PLCG1,PXDN,AJAP1,CEP85L,GAS2,CBX1,XG,SFXN1,BCL7A,HDGFL3,LY6H,PHGDH,FAM221A,GALNT2,GSPT2,NLGN4X,PEX5L,KDM5D,NUDT11,LAX1,RAB39B,MAGED1,CEP70,CACNB3,PRKCQ-AS1,SEC31B,RPS4Y1,DGKA,ITM2A,C12orf57,CD5,USP9Y,S1PR1,MARCKSL1,KIF15,ARHGEF3,YPEL1,KBTBD6,N4BP2,PGGHG,LAMP3,ZNF329,CD96,OR7E14P,CR2,LINC01806,SEPTIN6,PCBP3,NSG1,RCBTB2,ZBTB8A,UMODL1,EFCAB7,PTPRD,ZNF232,TET1,PRXL2A,LOC101928963,ASPM,RRAS2,SSBP2,TENM1,TESPA1,ANKRD36B,LUC7L3,ZNF260,C8orf88,LIMS2,LOC101928000,BUB1B,FGFR1,TCTEX1D2,PCNX2,PPP1R16B,CD28,MND1,E2F7,TBC1D31,ZNF101,LINC01120,SOX4,FAM111B,DPY19L2P2,SNHG19,C11orf80,PAXIP1-AS1,RAG2,CD27,PRKCH,ZNF738,MSANTD2,TMEM267,CCDC138,STMN1,PGM2L1,DDX5,HES1,RUNX1-IT1,TCF12,GPR174,FCGBP,MAGEH1,HMHB1,CEP128,PITPNM2,HELLS,RAP1GAP2,LCT-AS1,ADAM1A,BEX3,ARHGAP32,ZNF493,EPM2AIP1,CDH2,SH3BP5,VANGL2,PALLD,CBFA2T3,BARD1,CDK5RAP3,B4GALT6,ZNF439,SYNE2,LPAR6,SLC16A10,HMGCS1,NEIL3,KANK4,UGP2,IRF1-AS1,BAG3,H3C2,PGAP1,PLCL1,SMPD3,LZTFL1,PCDH9,TIAM1,MTA3,RIMS3,PAFAH1B3,ARMCX1,CAPSL,NREP,VANGL1,SIX6,ZNF415,SGPP1,MELK,MYL6B,TTK,SHQ1,ARHGAP21,STK39,DPEP1,FAM24B,KANSL1,ZNF767P,TMEM237,PCSK5,SLIT1,RFX7,MAGEE1,ADAMTS17,MIR181A2HG,RNF150,MGAT4A,KCTD1,KLF12,CD47,ADGRG1,MCUB,TBC1D19,KATNAL1,IRF2BPL,CDC25B,SLFN5,TRG-AS1,CNOT6,MFHAS1,SHPRH,ABCD3,ADGRB2,MYO7B,CLDN5,NUF2,ZBED6CL,STAMBPL1,ELOVL5

Supplementary Table 6. DEGs of AML vs T-ALL identified in GSE13204

Gene symbol (down-regulated DEGs)
ANXA5,PPT1,ENTPD1,SNCA,SERPINB8,LHFPL2,LINC01410,CPEB4,SCPEP1,ITGA5,SLC35D2,CETP,MXD1,TPST2,PLXNB2,ADGRA2,PDIA3,CPQ,LOXL3,RBKS,APOBR,ATP10D,GABARAPL1,ICAM1,PDIA6,SETD9,HOXB2,SH2B3,ACSM3,ADGRE5,NKG7,NCEH1,FNDC10,STING1,OAT,DOK3,DENND3,C5AR1,AHR,TRIB1,MARCHF2,IRF7,STEAP3,RXRA,THEM4,CD163,PLD1,HACD4,BIK,TMOD1,IFI27,CD34,TMEM216,NAMPT,CNN2,LY75,C1QB,CITED4,VMP1,LXN,ZFP36,PILRA,PLOD1,PIK3CB,HDHD5,TM6SF1,FAM49A,TMEM9B,AGTRAP,LOC100507642,FCER1A,SWAP70,IGSF10,SLC7A7,PLPPR3,SMIM20,DHX32,CLEC12A,NABP1,LRG1,TNFRSF1A,EPX,COL23A1,EMILIN2,P4HB,CPPED1,ALDH3A2,CLIC2,RALB,GBP2,ZSCAN9,CBR1,ZBTB16,VKORC1,KCNQ5,MSRB1,HSPC324,PNPLA4,CNRIP1,ZNF296,IRF8,FHL3,PIM3,STRADB,NFKB1,CEBPB,ATP6V0D1,PTAFR,CARD6,RELB,CRIP1,GATA2,GPT2,DENND1A,NACC2,NDRG1,FCN1,CAVIN2,LGALS2,JDP2,RAB7B,TYMP,VRK2,GPR160,NUDT19,DTD1,TMX4,HDC,CD180,PPIF,SELENOK,PCTP,LGALS12,TENT5C,GALNT1,P2RY13,ICAM4,EPHX1,NAPSB,B3GALNT1,RNF135,METTL7B,AREG,CCND2,SLC2A5,TNS3,EVA1B,ECPAS,COTL1,CCDC189,SIRPA,TET2,APOBEC3B,SELENON,NEU1,NIBAN1,H2BC21,DIAPH2,AOAH,SLC2A3,SGK1,PLAC8,GGTA1P,SNX9,VSIG4,KLHL2,SELENOP,PLEKHO2,HAL,ADAM28,CYSLTR1,NRIP3,LINC01943,PREX1,ALCAM,DHRS9,SERPINB1,C11orf74,MAP3K1,KCNE3,ARPC1B,KLF1,C9orf43,SUMF1,WFDC1,RHBDF2,PROSER2,CTSD,CD151,ADAP1,DYSF,TP53INP2,SPTLC2,IFNGR1,CAT,TBK1,CXCL3,JUP,TSPAN32,ANXA1,SDSL,SELENBP1,MANBA,PLP2,LMNA,MCRIP2,CD74,ELF4,MN1,CITED2,SLC43A1,PLOD3,MGAT4B,RRN3P2,CTSL,ATP2B1,DPEP2,HOXA10,CXCL12,BCL2A1,HSH2D,CPNE2,ROGDI,KCNE5,HCST,PCBD1,LYL1,S100P,ITGAV,SERPINB10,EREG,TSPAN4,KLF3,CD86,RGS2,PXK,ZSWIM6,IL3RA,1-Mar,ACTN4,MGST2,ZNF804A,PFKFB3,GBGT1,GRB10,DEFB1,FBP1,MAP3K8,GOLIM4,IL1RAP,FAM107B,JCHAIN,PHACTR1,ZEB2,GGH,KLF10,TNFSF13,SPNS2,PLD3,NOD2,ELL2,STAP1,ATP6AP1,SLITRK4,CCL5,GPD1L,CYBRD1,JUNB,M6PR,EGLN1,PLXDC2,ZNF516,VAMP8,CACNA2D3,LAMP2,MAN2B1,CPD,SNTB1,SYNGR1,FCHO2,BSG,S100A10,PYCARD,CCR1,CD14,RAB27A,MAFF,FAM210B,NFKBIE,SKAP2,SPINK2,LPCAT2,STOM,IGF2BP2,FBXO6,UPP1,S100A11,GPR27,FOSL1,FOXC1,VSIR,TLR4,TNFRSF1B,SCO2,P2RY2,DYNLT1,ARSD,ASAH1,IL10RA,HGF,TRH,CD55,LILRA2,S100A4,GMPR,NLRP3,SMIM3,TRIM58,MFSD10,CD44,UBE2E3,B3GNT5,TPP1,WDFY1,SLCO3A1,HSD17B11,SLC39A11,OGFRL1,DUSP5,ADM,LINC00623,NAGA,B4GALT5,C3orf80,SIRPB2,DEPDC7,GLA,PLBD2,CIDEB,PARVB,CSF1R,PTGER2,MROCKI,PSTPIP2,SPARC,BHLHE40,STAB1,GPAT3,DUSP23,CDKN1A,SLC27A3,ADGRE1,GNAI1,LDLRAD3,SPI1,EGFL7,RNASE6,CTSS,ZG16B,FUT4,IL6R,ABHD17C,CHCHD10,LTC4S,TFEC,PRSS21,TST,HMOX1,MAP7,HLA-DQB1,DUSP3,ANXA4,CST3,PNP,HEXB,EPS8,CLU,VCAN,CTSW,PRG2,BABAM2-AS1,JAG1,GLIPR1,C3AR1,KLF11,NR1H3,CTNNA1,SLC35F2,RASSF2,TSPO,LOC643072,MEF2C,HLA-DPB1,PIK3AP1,SPATA20,GNS,HYAL3,OSCAR,RILPL2,PTGS1,DUSP6,RAB31,SLC25A43,FGL2,PDE4B,NPR3,ID2,GCA,IL1B,SLC27A2,BST1,ERLIN1,CD63,ASL,MS4A7,NPW,PRKAR2B,NPC2,NKX2-3,IMPA2,CD36,MS4A6A,SAMHD1,TLR1,BLVRB,XK,PTH2R,CXCL8,MAP3K5,CTBP2,EGR1,OLIG1,MSRB3,PF4,ERMP1,CYFIP1,VSTM1,CCL23,GPX1,LMO2,HLA-DMB,FOSB,FOS,NFE2,NFIL3,SRGN,ATP2B1-

AS1,MAN1A1,CLEC5A,SPOPL,SUCNR1,LAPTM4B,GSAP,SLC22A15,TTC7B,MYO1F,NCF2,PIWIL4,STX11,TMEM220,MPEG1,PPP3CA,SDC4,PLAUR,HEBP1,MYADM,SRXN1,SLC31A2,LATS2,TIMP1,RAB13,FNDC3B,C17orf58,PRKCD,CTSB,IRS2,SERPING1,HCAR3,METRNL,CYTIP,IL13RA1,CLC,PROM1,KRT18,TENT5A,NR4A2,HLX,PRRG4,PLAGL1,RGS18,GTSF1,PLTP,IMPACT,ZNF185,TRBV27,PTGS2,FOSL2,TREM1,PLA2G4A,MEST,HOXA5,MFSD1,CST7,SLC22A4,FCER1G,ZYX,ACSL1,HOMER3,MEIS1,SERPINB2,MLC1,ARL4A,HCK,THEMIS2,FUCA2,GALNT11,PRAM1,RIPK2,CFP,LY86,IRAK3,APLP2,LGALS1,CD300LF,GRN,ADRB2,KLF4,MAMDC2,LYZ,BTK,LOC101928429,STAR,LOC105377276,CTSG,IGFBP7,HHEX,GAPT,NRGN,CA2,PDGFC,C1QTNF4,PLEK,PYGL,GPR183,CTSA,CEBPD,CREG1,LYN,CSF3R,NCF4,PROK2,MNDA,TLR2,TPSAB1,MS4A3,C1orf162,CSF2RB,PPBP,DRAM1,TYROBP,RNASE3,ECRP,LRMDA,FES,PRTN3,HLA-DPA1,F13A1,FCGR1B,ELANE,PTX3,CD33,CSTA,HLA-DRA,CCNA1,IL18,FLT3,PRXL2C,KIT,CYTL1,HLA-DMA,MGST1,PDLIM1,CEBPA,RNASE2,CPA3,TNFSF13B,TPSB2,TUBB6,MPO,ATP8B4,AZU1,CFD

Supplementary Table 7. DEGs of AML vs T-ALL identified in GSE51082

Gene symbol (up-regulated DEGs)
LCK,CD3D,TRBC1,MAL,RASGRP1,BCL11B,TSPAN7,PGGHG,CHI3L2,DNTT,SCN3A,ITM2A,CD7,LAT,MZB1,IL32,MLLT11,CD2,LTB,TRAC,TRAT1,FAT1,CD3E,LBH,ZAP70,RCBTB2,ENO2,TCF7,EVL,PVRIG,RHOH,CD247,LEF1,KRT1,USP20,TRIB2,GPRASP1,GATA3,ITK,CD1E,TUBB2A,SH2D1A,SYNE2,ARL4C,ADA,ELOVL4,GALNT6,STAM,DENND2D,MARCKSL1,SPRY1,FZD6,CD1B,UBASH3A,TXLNGY,CYFIP2,DGKA,FXYD2,EPHB6,PRKCQ,HELLS,MFHAS1,SOX4,CRY1,SELENOW,TCFL5,AEBP1,LPAR6,ST18,TOX,ZNF107,ADGRG1,TENM1,DBN1,TRAF5,ZNF711,CD28,ZNF91,INSIG1,LINC00342,LZTFL1,NINL,ELOVL5,CHD1,PIK3C2B,JAM3,CCR9,HMGCS1,USP9Y,SIX6,TBC1D4,TTK,TBC1D31,SEPTIN6,NDST3,ZNF423,ATP2A3,STMN1,CD1A,CD3G,TCEAL9,DHCR24,SERINC5,CBFA2T3,PTBP2,PKIA,CD8A,PTPRK,HMGCR,ENOSF1,NPTX2,ARPP21,TCF12,BEX3,ASPM,TTF1,MSMO1,KDM5D,CDK5RAP3,DDIT4,PPP1R16B,LINC01260,SLC4A8,MMD,MREG,CXADR,ZEB1,CLUHP3,BARD1,SH3BP5,SLC5A3,NOTCH1,HPGD,SCAI,CAPRIN2,EZH2,ACVR2B,ABLIM1,CR2,TMEM131L,ARID5B,DPY19L2P2,GNA15,HNRNPA1,ZNF331,ECT2,FUT8,CBX1,ZNF529,POU2AF1,CEP55,ABCD3,P2RX5,NLGN4X,CDC25B,GRAMD4,ITGAE,IL7R,ZSCAN18,LDLRAD4,SIT1,SEC31B,CD96,PRKCH,SSBP2,HDAC4,RUNX1-IT1,TASP1,SRSF11,CD2AP,CD6,DMTF1,SLC38A1,CCND3,TRAPPC10,EPM2AIP1,BDH2,DSTN,VAT1,NUP58,KRT2,C11orf80,SCD,CLK1,NAXD,HIVEP2,ALDH1A2,PCBP3,NOL11,NMT2,SLAMF1,BUB1B,PTPRCAP,PPM1A,ATXN7,MRTFB,NMRK1,SPTAN1,FBXO21,SH3GLB2,TSPYL4,CNOT6,MCUB,TCERG1,DDX39A,TSHR,FMR1,ACAD8,MSL2,ZBTB20,DSG2,ANKRD10,ZMYM2,PIK3R3,MYH10,SEPHS1,CSRNP2,CCDC92,PHGDH,SPTBN1,KDM3A,BTN3A2,PNMA1,PTPN3,KIF15,ARMCX2,SH2D2A,NSD2,EZR,MYL6B,KLRB1,CD27,ARHGEF7,PBK,ZNF135,CLGN

Supplementary Table 8. DEGs of AML vs T-ALL identified in GSE51082

Gene symbol (down-regulated DEGs)
CDKN1A,TALDO1,FBP1,GAA,DUSP1,CD164,TMX4,ERP29,MXI1,LRPAP1,HSD17B4,ADGRE2,IL13RA1,KCNN4,FOSB,HLA-DQB1,CES1,PRCP,SNAP29,HOXA10,DPEP2,PPA2,METTL7A,CST7,XPNPEP1,GSTO1,CD55,CAP1,KLF1,C5AR1,TGIF1,RGS19,SYNGR2,LAPTM4A,BSG,TNFRSF1B,SIPA1,TDRD7,PPT1,LIPA,CPA3,TOR4A,ASL,NKG7,SEC11A,MSRB1,ATP6V0D1,GLA,ID2,CCR1,TBXAS1,INPP5D,RAB27A,ASAH1,PDIA6,DEFA4,ZFP36,IRF7,STOM,GALNT11,CLU,PGD,CSF1R,ARHGEF40,FCGR2A,KLF11,GYG1,RBM47,AOAH,PLP2,ELK3,EGFL7,MRPL33,XBP1,LMNA,CD163,CCND2,ITGB2,LYST,TST,SERPING1,SLC31A2,IRF8,ECPAS,SPTLC2,AHNAK,TLR4,RAB9A,ADRB2,CTSC,FAH,HK3,CYFIP1,MEST,CTBP2,LAMP2,HLA-DMB,CTSW,SIRPA,S100A8,RPS27L,CTSD,GSTK1,CPD,LYL1,EVA1B,CTNNA1,LTBR,PNP,TIPARP,MGST2,PRSS21,CDA,PLCG2,ZNF185,DUSP3,MAP3K5,IDH1,SYK,CASP1,SNX3,CCNA1,ARL4A,MCTP1,DENND5A,CLEC11A,SLC27A3,CXCL8,ALCAM,NR4A2,SNX2,PLXND1,F13A1,WDR41,S100P,SKAP2,PSTPIP2,ADGRE5,C3AR1,MCL1,MGST3,CD44,STAR,EVI2B,CD93,PTGS2,GOLGA8N,LXN,C1QA,M6PR,TNFRSF1A,ACTN1,PLD3,PRG2,FCER1A,RAB31,TPP1,FTL,CTSS,PDIA3,BST2,MS4A4A,S100A12,CYTIP,GNS,LGALS2,B4GALT5,PRKAR2B,LITAF,FUT4,SELENOP,VNN1,TBK1,HLX,MAN2B1,ECRP,MFSD10,TUBB6,LEPROT,RAB13,MEF2C,TNS3,LY86,RASSF2,PTGER2,PLTP,MYO1F,GALNT1,RNASET2,MAN1A1,PPBP,PLAGL1,PROM1,STX11,CAT,CD9,RGS2,CSF2RB,GSAP,SAMHD1,CD36,BST1,CLC,CD33,ARL6IP5,S100A6,DYNLT1,CYTL1,KLF10,LILRA2,CTSB,ZEB2,BTK,IFNGR1,GSN,CYBA,MS4A6A,MLC1,BLVRB,KCTD12,PRKCD,NRGN,FCN1,C11orf21,ERLIN1,TLR2,CEBPB,NFE2,HHEX,HOMER3,KLF4,COQ2,PDLIM1,TPSAB1,NFIL3,TSPO,IMPA2,ANXA1,IGF2BP2,RNASE3,FCGR1B,FCER1G,CD14,ANXA4,TIMP1,S100A11,PPP3CA,NCF2,ZYX,HLA-DPA1,DRAM1,FLT3,CD63,BASP1,ACSL1,LYZ,PRTN3,PLEK,IRS2,S100A9,FOS,DUSP6,SERPINB1,VCAN,HEXB,LGALS3,PYCARD,LYN,GCA,STAB1,FGL2,THEMIS2,NPC2,EAF2,PDGFC,FES,IRAK3,SCPEP1,CFP,VAMP8,HEBP1,CTSA,CITED2,PYGL,APLP2,RNASE6,TNFSF13,CEBPD,MFSD1,SRGN,IGFBP7,CA2,HLA-DPB1,HSD17B11,TPSB2,S100A4,GRN,HCK,GLIPR1,LMO2,KIT,GPX1,TFEC,LGALS1,SPI1,MS4A3,HLA-DRA,CREG1,CEBPA,CTSG,CD74,SERPINB6,TRIB1,FNDC3B,PLAC8,HLA-DMA,CSF3R,NCF4,TENT5A,MNDA,CST3,ELANE,CSTA,MPO,ATP8B4,TYROBP,RNASE2,CFD,AZU1

Supplementary Table 9. DEGs of AML vs T-ALL identified in GSE89565

Gene symbol (up-regulated DEGs)
CTHRC1,CD3D,GXYLT2,SNTG2-AS1,FZD6,GAS2,CCR9,CHI3L2,LCK,ZNF711,CD7,ZAP70,GATA3,HHIP-AS1,TRBC1,TRAT1,PLCB4,TOX2,TSPAN7,MAL,BCL11B,MZB1,ELOVL4,PRTFDC1,FAM110C,SHISA2,FAM169A,HHIP,LOC285097,RASGRP1,DNTT,CD3G,SPRY1,CAMK4,LINC01215,SCN2A,ZNF827,CRNDE,ITK,NDST3,IFI44,FRMD6,CD3E,RHOH,ATP9A,SH2D1A,USP9Y,WAKMAR2,UBASH3A,MYEF2,ARPP21,TCF7,PCAT18,STARD4,TOX,PPP1R16B,CLGN,P2RX5,ARMCX2,LUNAR1,ICOS,RUFY3,FAT1,ARL4C,NID2,ZBTB8A,IFI44L,CRY1,MME,XG,H2BW4P,UMODL1,SLC19A2,LEF1,HES4,SCAI,IL7R,CD247,PGAP1,STAT4,C12orf75,TASP1,KDM5D,N4BP2,SBK1,VPREB1,KIF3A,TUBB2A,TRIB2,GJA1,SCN3A,ITM2A,CYFIP2,LEF1-AS1,SEPTIN1,NMT2,LAX1,TMEM131L,GPRASP1,TENM1,GALNT6,PGGHG,KRT72,CHD1,CLDN5,LOC101928963,CHRNA5,CAPSL,SEC31B,MLLT11,PIK3C2B,AMIGO2,CD1B,SCGB3A1,LZTFL1,ZNF682,RPS4Y1,PXDN,DBH-AS1,LINC01238,PVRIG,CDH2,CEP85L,ENO2,IFITM1,DUXAP10,MIR646HG,SH3BP5,AEBP1,SFXN1,TTTY15,FCGBP,TXLNGY,C11orf80,ALMS1-IT1,PTPRK,RUBCNL,EVL,MREG,PTPRD,SERINC5,KCNK17,LIPC,PJVK,RAP1GAP2,DDIT4,ALDH8A1,ZBTB21,CD96,GALNT2,CXorf65,LAT,GPR174,PRKCH,TXK,FXYD2,ADAMTS1,SPAG1,TNFAIP3,BACE2,SLC7A3,HAUS3,ERV3-2,CR2,IL32,BTBD11,RANBP2,PCDHB14,LINC01806,TRO,CLK1,RFPL3S,DPEP1,PRKCQ,PABPC4L,PMAIP1,GBP1,LDLRAD4,NBAT1,SLC37A3,SLC4A4,NSG1,MORC4,RHOBTB1,NAP1L5,SMIM17,ZEB1,MAP4K3,ELOVL5,LINC01260,ENPEP,RNF103,PTBP2,CCDC138,EPSTI1,ARHGAP5,EIF1AY,HES1,NECTIN3,DGKA,PRSS2,RORA,RAB15,PCDH10,HMHB1,PXYLP1,TSHR,CCNL1,LRP12,ABLIM1,TFDP2,LPAR6,CENPU,CNN3,TMEM178A,NUCB2,ERVFRD-1,SLC4A8,RTL6,ZNF57,PIGA,EPHB6,VASH2,DPP4,HMGCS1,LGALS3BP,SLC7A11,CCDC136,BAG3,LCT-AS1,CCDC141,TIAM1,ARHGAP21,MGAT4A,LOC100505501,TSPYL2,NINL,S1PR1,THUMPD2,CEP95,PLCG1,CLCA1,CEP70,CDCA7,SLC30A4,FGFR1,LOC105375172,MOB1B,STAM,NLRC3,WRN,PLCG1-AS1,KLF12,VANGL2,DDX3Y,RGS9,LINC00597,RCBTB2,SH2D2A,GORAB,TESPA1,HNRNPA1,LRP2BP,YOD1,OCIAD2,PLEKHA7,TNF,TBC1D32

Supplementary Table 10. DEGs of AML vs T-ALL identified in GSE89565

Gene symbol (down-regulated DEGs)
C1QTNF4,LRRC25,UCP2,TRIM6,PSD3,CKM,RAB32,LTC4S,MGAT4B,BAX,C2orf76,CD93,ORMDL2,AHSP,CPVL,PTGER2,RRAGD,SELENOP,PIWIL4,ASAP2,SERPINB2,PSENEN,GYG1,ANGPT1,DYNLL1,PIK3CG,HHEX,TLR1,HMOX1,SDC4,H2BC12,FGD4,LCN2,HOXB3,PEAR1,DPY19L2,FKBP9,JAML,TMA16,PSTPIP2,CCL5,MILR1,MRPS21,TNFAIP6,CLCC1,P2RY2,CTSD,PNPLA4,MRPS28,THEM4,SIPA1,PLD3,METTL7B,LINC01410,TNFRSF1B,CLIC2,PHF23,GLRX2,TNFSF13,ACVR1,KYNU,HOXB-AS3,CD37,CD24,MIR10A,CYTIP,ARHGEF40,HDHD5,TMEM71,TMEM106B,DEGS1,FAM210B,DHRS7B,TPK1,SH3BGRL3,LILRB2,RALB,DYNLT1,RBPMS,FCHO2,EGR2,CST7,GFI1B,ANXA3,CX3CR1,TRIB1,SEPTIN11,NDUFV3,MUC19,LRRK2,EGFL7,RHBDF2,BASP1,TMTC2,BRI3BP,SLC2A5,SHTN1,CERS6,LINC00899,LGALS2,CCDC126,HBB,RASSF2,C1GALT1C1,GNS,CYBRD1,IGF2BP2,HPCAL1,MVP,NCEH1,RILPL2,DERA,MAP3K1,RTL8C,ZC3H12C,STK32B,IGHM,VASP,TIMP1,ZNF667-AS1,ITGA9,IL10RA,GPN3,NACC2,ADORA2B,DOK3,RNF135,H2BC21,VNN2,FOS,LDLRAP1,FCGR3B,LRG1,NAPSB,TNFRSF17,ATP6V0D1,OLFML2A,TST,PAM,TET2,PPP1R3D,KRT18,LOC339803,SRGAP2C,RASSF3,ATP6V1A,CPD,PNP,TMEM216,ZDHHC2,CAVIN2,MAN1A1,APPL1,TNS3,RNF217,NIBAN1,LRRK1,JAG1,RASGRP4,SLC35D2,HOXA10,IRF2,IGSF6,PRKAR2B,NLRP3,ACSM3,EPDR1,ATG4C,MLC1,LPL,ARL4A,B3GNT5,PHACTR1,CD86,STAP1,RRAS,ZNF296,SIRPA,FCER1A,PTAFR,DCAF12,KIAA0930,PCTP,RIPK2,LYST,SIGLEC5,LINC01181,PF4,KIF13A,NABP1,S100A4,LGALS1,APOBEC3B,CETN3,TSPO,SNTB1,QKI,ENTPD1,HCAR3,NOD2,MARCHF1,SYK,MYOF,C17orf58,ARPC1B,LGALS3,AP2B1,HPSE,STX3,SETD6,HLA-DQA1,PTGR1,ADAM28,STX7,NPC2,S100A10,HCST,LOC100996740,C5AR1,B4GALT5,MS4A4A,CSF2RA,STOM,NOG,RGS19,VSIG4,SERPINB10,ALDH3A2,PLXDC2,PIP5K1B,ERMP1,GASK1B,RGS2,PARVB,CTSB,FUT4,TMEM60,IGFBP7,BAALC,SMIM20,MGAM,SLC22A15,PLBD1,PLTP,MINPP1,EPX,SKAP2,TMEM220,ZEB2,LYL1,CISD1,GPR183,SULF2,IGSF10,CD44,SPTSSA,CD74,CREBL2,HYMAI,CTSA,BSG,GPD1L,TENT5A,SPOPL,CYSLTR1,IRS2,VAMP8,FNDC3B,SDSL,TPP1,CPPED1,FUCA2,PTPN18,STX11,FCGR2B,MGST2,FRAT1,OSCAR,FPGT,SRXN1,SLC7A7,SPATA20,SLCO3A1,DUSP3,FAM107B,C14orf119,SRGAP2,SNAI3,CD180,C11orf74,SLC45A3,ABHD15,CNRIP1,ERLIN1,TRIM58,PROM1,PLD1,HSPC324,GBGT1,SERPING1,CD36,MCTP1,NAGA,KBTBD11,NRGN,ZNF185,ADGRE1,CTSS,PTGS1,CST3,VSIR,DIAPH2,MMP9,CPNE8,HLA-DQB1,TNFRSF1A,KLF1,GLIPR1,BLVRB,FCN1,ITPRIPL2,QPCT,HK3,XIST,TLR8,CIDEB,FRAT2,LOC100507642,HEATR5A,MEF2C,CXCR2,CRISP3,ASL,MN1,PTGS2,BST1,VCAN,CYFIP1,HGF,HLA-DPB1,WDR49,HEBP1,C5orf30,SLC22A4,FAR2,CARD8-AS1,CHCHD10,DUSP23,M6PR,CLEC12A,PRR34-AS1,GGTA1P,HOXA5,LY86,GGH,MAP3K5,MSRB3,TREM1,HOMER3,NRIP1,PLAGL1,ARSD,CLEC5A,RAB7B,CDA,LOC643072,CCNA1,CPNE2,PTX3,LXN,S100A11,FAM49A,TTC7B,HLA-DMB,S100P,CREG1,MROCKI,SLC27A3,ZYX,P2RY13,ITGAV,CD14,ACSL1,EGLN1,LYN,EGR1,THSD7A,S100A8,LILRA2,CCR2,CEACAM6,NKX2-3,ANXA4,KCTD12,GPR160,RAB13,MMRN1,FGR,PPP3CA,APLP2,HOXB2,TFEC,ANXA5,TLR4,LOC105377276,ZNF516,UBE2E2,NPR3,MFSD1,ATP10D,ZNF804A,RAB31,KCNE3,PRRG4,O

LIG1,CBR1,TNFAIP8L2,C3AR1,SLITRK4,PIK3AP1,FGL2,LACTB2,EPS8,LRMDA,LINC00623,CARD6,NR1H3,CACNA2D3,SLC27A2,MS4A6A,VSTM1,KLF4,BCL11A,GRB10,CTNNA1,CSF1R,MSRB1,CRIP1,PYCARD,RGS18,THEMIS2,GPX1,GSAP,SPI1,CD300LF,CA2,CCL23,MYADM,S100A12,PLEK,DHRS9,PRAM1,FCER1G,MANSC1,MMP8,DRAM1,TRBV27,SRGN,FES,DEPDC7,PRXL2C,SERPINI2,CCR1,GALNT11,PRAG1,CTBP2,RXFP1,SUCNR1,GRN,IMPACT,HBD,CTSG,NFE2,GCA,LTF,GTSF1,MYO1F,B3GALNT1,S100A9,BLNK,IMPA2,HLX,IL6R,STAR,FLT3,MS4A7,BTK,MEIS1,P2RY14,SAMHD1,SLC25A43,DACH1,CYTL1,ADRB2,C3orf80,SIRPB2,CSF2RB,TCN1,GPAT3,MAP7,CEBPD,IL1B,LMO2,MEST,SMIM15,CPA3,CSF3R,IRAK3,PTH2R,DUSP6,RNASE6,PPBP,IL13RA1,HLA-DRA,ZSWIM6,PROK2,PRG2,PRKCD,UBE2E3,TPSAB1,C1orf162,NCF4,ECRP,CD33,KIT,F13A1,NCF2,CEACAM8,PYGL,LYZ,PDLIM1,VNN1,HLA-DMA,MPEG1,HLA-DPA1,HCK,CFP,MAMDC2,DEFA4,PRTN3,PDGFC,RNASE3,TLR2,TNFSF13B,FCGR1B,TUBB6,GNAI1,TYROBP,LOC102724587,CSTA,CEBPA,IL18,MS4A3,CLC,LOC101928429,MGST1,MNDA,TPSB2,GAPT,PLA2G4A,MPO,ELANE,CFD,ATP8B4,RNASE2,AZU1

Supplementary Table 11. DEGs of AML vs T-ALL identified in GSE131184

Gene symbol (up-regulated DEGs)
CTHRC1,GXYLT2,CD3D,FZD6,CHI3L2,MAL,HHIP-AS1,LCK,ZNF711,SNTG2-AS1,CCR9,TOX2,CD7,GATA3,PLCB4,GAS2,ZAP70,CD3G,TRBC1,PRTFDC1,TRAT1,CAMK4,HHIP,ELOVL4,TSPAN7,SHISA2,FAM110C,BCL11B,FAM169A,LINC01215,MZB1,DNTT,CRNDE,WAKMAR2,SH2D1A,RASGRP1,SCN2A,ZNF827,SPRY1,LOC285097,UBASH3A,ICOS,ITK,CD3E,FRMD6,LEF1,ARPP21,NDST3,FAT1,TCF7,PCAT18,TUBB2A,SEPTIN1,TOX,MYEF2,RHOH,CLGN,PPP1R16B,SCGB3A1,HES4,H2BW4P,CRY1,ARL4C,P2RX5,NID2,RUFY3,CD1B,LEF1-AS1,SBK1,MLLT11,KRT72,SCAI,MME,STARD4,RPS4Y1,ARMCX2,CLDN5,IRX5,ATP9A,PVRIG,TRIB2,BAG3,VPREB1,KIF3A,CYFIP2,USP9Y,EVL,IFI44,PXDN,SLC19A2,CD247,LUNAR1,TASP1,CAPSL,MYH10,NMT2,PGGHG,PABPC4L,KCNK17,TMEM131L,KDM5D,GALNT6,CDH2,XG,C12orf75,JUN,LINC01260,CXorf65,UMODL1,PGAP1,GPRASP1,FCGBP,SFXN1,SCN3A,ITM2A,DDIT4,LAT,IL7R,STAT4,TNFAIP3,AEBP1,IFITM1,DBH-AS1,PMAIP1,LZTFL1,LINC01806,SH3BP5,LOC101928963,ZEB1,SEC31B,ABLIM1,DPEP1,H3C2,LRP12,CHRNA5,FXYD2,PIK3C2B,CHD1,LDLRAD4,DUXAP10,OCIAD2,TENM1,HES1,N4BP2,ARHGAP21,PARD3,LIMS2,TXLNGY,BCL7A,ENO2,ALMS1-IT1,LINC01238,SLC7A3,PBK,RAP1GAP2,PTPRD,CD1E,CD1A,PKIA,LAX1,MREG,CEP85L,TSPYL2,HAUS3,RAB15,CR2,NAP1L5,NBAT1,PCBP3,EPHB6,RORA,PRKCH,BACE2,LIPC,CENPU,HMHB1,GALNT2,RUBCNL,TTTY15,CLK1,PIGA,ZBTB21,ZNF682,ENPEP,ZNF423,RNF103,SLC37A3,TSHR,PLCG1,TFDP2,TIAM1,NINL,PRKCQ,TMEM178A,NSG1,WHAMM,ARHGAP32,MSL2,RGS9,NKD2,PEX5L,TRO,EIF1AY,PXYLP1,RTL6,BTBD11,PCDHB14,SLC23A1,GPR17,ARHGAP5,YOD1,CD2,ALDH8A1,RCBTB2,ZNF329,HNRNPA1,TNF,HDGFL3,ELOVL5,MORC4,SELENOW,ZBTB8A,LCT-AS1,KLF12,CCNL1,OR7E14P,PCDH10,GAL3ST4,SLC4A4,SERINC5,CEP95,CCDC136,STMN1,VASH2,PLCL1,KCNA3,RLF,PRSS2,LOC101928000,IFI44L,FHIT,ZNF836,CCDC141,CD5,PJVK,LY6H,GPR174,DPP4,LOC100505501,PLEKHG4B,NLGN4X,LIME1,MOB1B,DDX3Y,MEX3C,BEX3,SALL2,SLC16A10,IL32,AJAP1,CCDC138,STAM,CEP70,SCG2,ADA,NLRC3,MGAT4A,SLC7A11,SMIM17,MAGED1,RHOBTB1,RANBP2,H1-3,THUMPD2,CDCA7,LPAR6,EPSTI1

Supplementary Table 12. DEGs of AML vs T-ALL identified in GSE131184

Gene symbol (down-regulated DEGs)
HOXB-AS3,TRIM27,SHTN1,NETO2,GLCE,KCNQ5,DYSF,IDH1,RTL8C,NRIP1,CRNKL1,HAL,SMCO4,ATP2B1,HIP1,OLFML2A,RNASEL,MMEL1,RBKS,NIBAN1,BASP1,AQP9,RHBDF2,CPT2,TCTEX1D1,GMPR,MAP3K20,CD37,ZDHHC2,SIGLEC5,ZNF296,TMTC2,MTLN,PDE4B,GLRX2,TNFRSF1B,GLA,HPS5,B3GNT5,TIMP1,SPART,TRIB1,ZSCAN31,METTL7B,CASP1,SYNGR1,LGALS2,C20orf194,IGHM,CRISP3,FAM174A,ATP6V0E1,SRGAP2C,ST3GAL6,PRKAR2B,PCBD1,MILR1,MRC1,DUSP27,PHACTR1,CD24,EPHX1,PTAFR,WFDC1,AGTRAP,GYG1,APPL1,LRG1,RALB,PPP1R3D,DEFB1,SCO2,CERS6,C17orf58,FGD4,SLPI,FRMD3,C2orf76,TST,MAP3K1,BCL2A1,RGS19,MRPS28,C1QTNF4,RHOQ,LY75,DERA,PLXDC2,BEX1,LOC100996740,COPS7A,EEF1AKNMT,LOC339803,IGSF6,MIR10A,RNFT1,GPR27,ELOVL7,ZNF404,DCAF12,NCEH1,HOXB3,INPP5D,ANGPT1,RNF217,QKI,ACVR1,RAB10,SELENOP,RPP40,S100A4,RASGRP4,SWAP70,ZC3H12C,NOD2,SLC35F2,HSD17B11,TSPO,LPL,CPD,TMA16,PRMT6,THEM4,BSG,BLVRB,ARPC1B,PLPP6,NDUFAF1,SPINK2,EGFL7,TMEM71,OPN3,ARL4A,STX3,SDSL,SPTLC2,STK32B,DOK3,DAB2,BBS12,CCPG1,SLC22A16,GNG11,ASAP2,CNRIP1,TMEM106B,PSTPIP2,LGALS3,NACC2,GOLPH3L,SIRPA,ARHGEF40,CCDC126,IRF2,TMEM192,DYNLT1,EGR3,PRR34-AS1,LDLRAP1,NAPSB,KIAA0930,DHRS7B,SLC22A15,SERPINB10,EGR2,CYTIP,DPY19L2,CD86,ATP6V1A,TMEM216,NLRP3,RAB32,GBGT1,RBPMS,PNPLA4,ANXA1,SERPINB2,MGST2,DYNLL1,IGF2BP2,SLC31A2,PHF23,RRAS,CXorf21,RASSF2,CAVIN2,HDHD5,BNIP3,JAK2,MGAM,PNP,CD34,SPATA20,PCTP,UBR5-AS1,TRH,ARL11,SLC35A5,IL10RA,PAM,FCER1A,ASL,PARVB,PIP5K1B,ANXA5,ABHD17C,STAP1,LAPTM4B,MUC19,CAV1,CLCC1,COCH,LYST,ANTXR2,LINC01410,ITGA9,RNF135,MYOF,RIPK2,SLC15A2,SNTB1,TNS3,FOS,PF4,TRIM58,VSIR,SLC35D2,SRXN1,DEGS1,HHEX,NPW,FUT4,TNFAIP6,VSIG4,MINPP1,VNN2,KIF13A,C1GALT1C1,JAML,CD44,LRRK2,SPARC,HK3,LINC00899,PTGR1,TNFRSF17,XIST,FCN1,ADGRE1,SMIM20,BRI3BP,EPX,ADORA2B,SERPING1,STX7,CPNE8,HLA-DQA1,CD74,MS4A4A,IGFBP7,FRAT1,SLC2A5,ENTPD1,ALDH3A2,SETD6,FAM107B,GASK1B,ADAM28,ANXA3,SLCO3A1,SNAI3,CSF2RA,SPTSSA,LYL1,CST7,IRS2,LINC01181,TPK1,ACSM3,CTSB,SULF2,GPN3,CDA,TLR1,PSENEN,STX11,HBD,KRT18,FUCA2,VAMP8,CETN3,GGH,SLC45A3,STOM,RGS2,MARCHF1,SLC7A7,PTPN18,BCL11A,IGSF10,MLC1,NABP1,CD36,KLHL13,LRRK1,PTGER2,CYBRD1,CPPED1,RASSF3,SYK,HPSE,CST3,CX3CR1,B4GALT5,ITPRIPL2,S100A10,FRAT2,PIWIL4,GPD1L,ABHD15,ZEB2,C14orf119,SRGAP2,SPOPL,CREBL2,FNBP1L,C5AR1,TMEM220,GPR183,ATG4C,HCAR3,NAGA,LGALS1,ZYX,CRIP1,EGLN1,QPCT,TREM1,DUSP3,GNS,OSCAR,TPP1,CTSA,TMEM60,HEBP1,FCGR2B,BAALC,M6PR,MCTP1,S100P,CIDEB,EPDR1,HLA-DQB1,MAN1A1,CHCHD10,TNFRSF1A,CA2,FCGR3B,PPP3CA,HSPC324,C11orf74,PLTP,ITGAV,FAM49A,PTGS1,GLIPR1,CYSLTR1,CPNE2,MROCKI,FAR2,WDR49,ERMP1,FPGT,OLIG1,CXCR2,UBE2E2,HLA-DPB1,SLC27A3,MN1,C5orf30,LY86,SKAP2,CEACAM6,NRGN,CTSS,MMRN1,LTF,HOXA5,KBTBD11,PLD1,GPR160,ARSD,BST1,LXN,DIAPH2,TENT5A,CARD8-AS1,CBR1,SLITRK4,FNDC3B,HOMER3,S100A8,GPX1,LOC100507642,S100A11,APLP2,SLC22A4,FGR,DUSP23,KLF4,HOXB2,ZNF185,HEATR5A,RAB13,PRRG4,LOC643072,RAB7B,TLR

8,LILRA2,PTX3,LINC00623,CYFIP1,NOG,TTC7B,HLA-DMB,LYN,CCL23,PYCARD,THSD7A,TLR4,ANXA4,GGTA1P,MYADM,LOC105377276,CARD6,ERLIN1,SPI1,CD14,HGF,MEF2C,CD180,CREG1,NKX2-3,TFEC,VCAN,NR1H3,EPS8,CTNNA1,MSRB3,PTGS2,GSAP,SLC27A2,CCR2,KCNE3,MSRB1,LRMDA,GRB10,S100A12,PIK3AP1,P2RY13,CYTL1,ACSL1,HLX,DACH1,HYMAI,MANSC1,ZNF516,MMP8,CLEC5A,CD300LF,CLEC12A,NPR3,RXFP1,MFSD1,PRAM1,CCR1,LACTB2,MAP3K5,IMPA2,THEMIS2,VSTM1,MAP7,KCTD12,PRG2,SERPINI2,CSF1R,GRN,CACNA2D3,CTSG,ZNF804A,TNFAIP8L2,MYO1F,PLEK,RAB31,UBE2E3,CCNA1,SRGN,CTBP2,B3GALNT1,ATP10D,PLAGL1,DUSP6,PRXL2C,DHRS9,C3AR1,FES,BLNK,PRAG1,MS4A7,MS4A6A,FGL2,FCER1G,STAR,RNASE6,CSF2RB,C3orf80,ZSWIM6,IL6R,NFE2,GPAT3,TCN1,S100A9,IMPACT,BTK,PROM1,EGR1,SMIM15,GALNT11,IL1B,CSF3R,MEIS1,CEBPD,DEPDC7,PPBP,GTSF1,IL13RA1,P2RY14,SIRPB2,PRKCD,CD33,IRAK3,PTH2R,DRAM1,SAMHD1,GCA,TPSAB1,DEFA4,NCF4,C1orf162,CEACAM8,LMO2,TRBV27,CFP,VNN1,ECRP,MEST,PDLIM1,ADRB2,RGS18,MPEG1,SLC25A43,NCF2,HCK,PROK2,LYZ,PRTN3,CPA3,HLA-DPA1,FLT3,HLA-DMA,HLA-DRA,GNAI1,MAMDC2,SUCNR1,PYGL,TYROBP,RNASE3,LOC102724587,TUBB6,CEBPA,TLR2,KIT,PDGFC,CLC,MS4A3,CSTA,IL18,F13A1,LOC101928429,FCGR1B,TNFSF13B,MPO,ELANE,TPSB2,PLA2G4A,MGST1,RNASE2,MNDA,AZU1,GAPT,CFD,ATP8B4

Supplementary Table 13. Top 5 GO Enrichment Analysis Results for the AML vs T-ALL comparison

Category	Term	Count	%	*P* Value	Genes
Biological Process(BP)	immune response	23	13.45029	1.00E-10	CCR1, IL32, CD74, CR2, C5AR1, PRG2, PPBP, CST7, SAMHD1, TNFRSF1B, CTSS, HLA-DMB, ZEB1, PNP, HLA-DPB1, HLA-DRA, CTSG, CD36, IL7R, TLR4, LAT, HLA-DPA1, HLA-DQB1
	inflammatory response	20	11.69591	4.14E-09	LYN, CCR1, CSF1R, LXN, PTGER2, C5AR1, LY86, PPBP, FOS, AZU1, LYZ, PTGS2, TNFRSF1B, THEMIS2, KIT, C3AR1, PRKCQ, CD14, TLR4, LAT
	response to lipopolysaccharide	11	6.432749	4.09E-06	PTGER2, C5AR1, CTSG, CSF2RB, FOS, PPBP, TRIB1, PTGS2, MPO, TLR4, ELANE
	T cell receptor signaling pathway	10	5.847953	1.29E-05	ITK, THEMIS2, TRAT1, HLA-DPB1, HLA-DRA, PRKCQ, CD247, LAT, HLA-DPA1, HLA-DQB1
	antigen processing and presentation	7	4.093567	1.39E-05	CD74, HLA-DMB, HLA-DPB1, HLA-DRA, CTSS, HLA-DPA1, HLA-DQB1
Cellular Component(CC)	plasma membrane	65	38.0117	3.20E-06	FLT3, MLC1, IRS2, ENO2, GLIPR1, RGS2, CA2, C3AR1, FCER1A, CTSG, HOMER3, CD36, HLA-DPA1, PPP1R16B, CR2, FCER1G, PRKCH, ACSL1, PVRIG, TNFRSF1B, ARL4A, KIT, BLVRB, PRTN3, PRKCQ, TLR4, CD44, HLA-DQB1, SKAP2, CSF1R, PTGER2, C5AR1, CSF2RB, ADRB2, SAMHD1, RASGRP1, CD1B, STX11, PRKAR2B, PDGFC, CD14, PROM1, SCN3A, CCR1, LYN, CD74, ZNF185, GCA, TRAT1, LY86, TSHR, BST1, LPAR6, CPD, FXYD2, RAB13, FAT1, HLA-DPB1, HLA-DRA, NMT2,

					CD247, IL7R, ITM2A, LAT, SERINC5
	cell surface	19	11.11111	2.55E-06	CD74, CSF1R, FCER1G, C5AR1, CD1B, TSHR, FUT4, LGALS1, PDGFC, HLA-DPB1, HLA-DRA, FCER1A, CTSG, CD36, PROM1, TLR4, CD44, ELANE, HLA-DPA1
	MHC class II protein complex	6	3.508772	1.41E-06	CD74, HLA-DMB, HLA-DPB1, HLA-DRA, HLA-DPA1, HLA-DQB1
	extracellular space	39	22.80702	3.08E-10	CFD, CSTA, AEBP1, CST7, ENO2, MPO, CTSS, CST3, LGALS1, CA2, PDGFC, STOM, CYTL1, IGFBP7, CTSG, CD14, TIMP1, CD36, PROM1, PLTP, ELANE, S100A11, CTSB, CPA3, IL32, EGFL7, LY86, RNASE3, PPBP, AZU1, LYZ, VCAN, TST, CPD, SELENOP, KIT, SERPING1, S100A4, PRTN3
	extracellular exosome	60	35.08772	1.70E-10	CYFIP1, ENO2, MPO, HEBP1, TUBB6, LGALS1, PNP, CA2, TSPO, MAN1A1, CTSG, TIMP1, CTSB, CR2, PRKCH, RNASE6, RNASE3, TST, SELENOP, BLVRB, SERPING1, ZNF711, S100A4, PRTN3, CD44, B4GALT5, CFD, CSTA, LXN, FGL2, AEBP1, GNS, CST3, PRKAR2B, PDGFC, STOM, IGFBP7, ASL, CD14, PROM1, MEST, S100A11, ELANE, LYN, CTSA, CD74, GCA, PRG2, AZU1, LYZ, BST1, CPD, FXYD2, RAB13, FAT1, HLA-DRA, S100P, MNDA, ITM2A, SERINC5
Molecular Function(MF)	protease binding	6	3.508772	0.002968	CST3, CSTA, KIT, TIMP1, CST7, ELANE
	peptide antigen binding	4	2.339181	0.002425	HLA-DPB1, HLA-DRA, HLA-DPA1, HLA-DQB1

	cytokine binding	4	2.339181	7.64E-04	CSF1R, CD74, KIT, ELANE
	cytokine receptor activity	5	2.923977	3.88E-04	CD74, FLT3, CSF2RB, IL7R, CD44
	MHC class II receptor activity	4	2.339181	3.69E-04	HLA-DPB1, HLA-DRA, HLA-DPA1, HLA-DQB1

Supplementary Table 14. Top 5 GO Enrichment Results for Upregulated DEGs Identified for the AML vs T-ALL Comparison

Category	Term	Count	%	*P* Value	Genes
Biological Process(BP)	T cell receptor signaling pathway	5	11.62791	3.75E-04	ITK, TRAT1, PRKCQ, CD247, LAT
	adaptive immune response	4	9.302326	0.004861	ITK, TRAT1, CD1B, LAT
	intracellular signal transduction	5	11.62791	0.013862	ITK, PRKCH, SH3BP5, PRKCQ, LAT
	regulation of phosphatidylinositol 3-kinase signaling	3	6.976744	0.014124	PPP1R16B, TRAT1, RASGRP1
	immune response	5	11.62791	0.016036	IL32, CR2, ZEB1, IL7R, LAT
Cellular Component(CC)	mast cell granule	2	4.651163	0.046222	RASGRP1, LAT
	T cell receptor complex	2	4.651163	0.039749	TRAT1, CD247
	cell-cell junction	4	9.302326	0.00677	ITK, PRKCH, FAT1, LAT

	plasma membrane	20	46.51163	6.71E-04	PPP1R16B, CR2, PRKCH, TRAT1, PVRIG, ENO2, RASGRP1, CD1B, TSHR, LPAR6, FXYD2, FAT1, NMT2, PRKCQ, CD247, IL7R, ITM2A, SCN3A, LAT, SERINC5
Molecular Function(MF)	protein kinase C activity	2	4.651163	0.033252	PRKCH, PRKCQ

Supplementary Table 15. Top 5 GO Enrichment Results for Downregulated DEGs Identified for the AML vs T-ALL Comparison

Category	Term	Count	%	*P* Value	Genes
Biological Process(BP)	inflammatory response	18	14.0625	1.86E-09	LYN, CCR1, CSF1R, LXN, PTGER2, C5AR1, LY86, PPBP, FOS, AZU1, LYZ, PTGS2, TNFRSF1B, THEMIS2, KIT, C3AR1, CD14, TLR4
	immune response	18	14.0625	8.99E-09	CCR1, CD74, C5AR1, PRG2, PPBP, CST7, SAMHD1, TNFRSF1B, CTSS, HLA-DMB, PNP, HLA-DPB1, HLA-DRA, CTSG, CD36, TLR4, HLA-DPA1, HLA-DQB1
	response to lipopolysaccharide	11	8.59375	2.99E-07	PTGER2, C5AR1, CTSG, CSF2RB, FOS, PPBP, TRIB1, PTGS2, MPO, TLR4, ELANE
	antigen processing and presentation	7	5.46875	2.69E-06	CD74, HLA-DMB, HLA-DPB1, HLA-DRA, CTSS, HLA-DPA1, HLA-DQB1
	antigen processing and presentation of exogenous peptide antigen via MHC class II	8	6.25	4.47E-06	CD74, HLA-DMB, FCER1G, HLA-DPB1, HLA-DRA, CTSS, HLA-DPA1, HLA-DQB1
Cellular Component(CC)	extracellular region	29	22.65625	3.38E-06	CFD, FCN1, F13A1, LILRA2, CTSS, HEBP1, CST3, GLIPR1, PNP, PDGFC, IGFBP7, CTSG, CD14, TIMP1, PLTP, ELANE, CTSB, CPA3, EGFL7, RNASE6, PRG2, RNASE3, PPBP, AZU1, TNFRSF1B, LYZ, VCAN, SELENOP, SERPING1
	cell surface	17	13.28125	9.40E-07	CD74, CSF1R, FCER1G, C5AR1, FUT4, LGALS1, PDGFC, HLA-DPB1, HLA-DRA, FCER1A, CTSG,

					CD36, PROM1, TLR4, CD44, ELANE, HLA-DPA1
	MHC class II protein complex	6	4.6875	3.36E-07	CD74, HLA-DMB, HLA-DPB1, HLA-DRA, HLA-DPA1, HLA-DQB1
	extracellular exosome	51	39.84375	2.51E-11	CYFIP1, MPO, HEBP1, TUBB6, LGALS1, PNP, CA2, TSPO, MAN1A1, CTSG, TIMP1, CTSB, RNASE6, RNASE3, TST, SELENOP, BLVRB, SERPING1, S100A4, PRTN3, CD44, B4GALT5, CFD, CSTA, LXN, FGL2, GNS, CST3, PRKAR2B, PDGFC, STOM, IGFBP7, ASL, CD14, PROM1, MEST, S100A11, ELANE, LYN, CTSA, CD74, GCA, PRG2, AZU1, LYZ, BST1, CPD, RAB13, HLA-DRA, S100P, MNDA
	extracellular space	36	28.125	2.98E-12	CFD, CSTA, CST7, MPO, CTSS, CST3, LGALS1, CA2, PDGFC, STOM, CYTL1, IGFBP7, CTSG, CD14, TIMP1, CD36, PROM1, PLTP, ELANE, S100A11, CTSB, CPA3, EGFL7, LY86, RNASE3, PPBP, AZU1, LYZ, VCAN, TST, CPD, SELENOP, KIT, SERPING1, S100A4, PRTN3
Molecular Function(MF)	cytokine receptor activity	4	3.125	0.002314	CD74, FLT3, CSF2RB, CD44
	peptide antigen binding	4	3.125	0.001108	HLA-DPB1, HLA-DRA, HLA-DPA1, HLA-DQB1
	protease binding	6	4.6875	8.90E-04	CST3, CSTA, KIT, TIMP1, CST7, ELANE
	cytokine binding	4	3.125	3.44E-04	CSF1R, CD74, KIT, ELANE

	MHC class II receptor activity	4	3.125	1.65E-04	HLA-DPB1, HLA-DRA, HLA-DPA1, HLA-DQB1

Supplementary Table 16. The DEGs with the top 10 highest network degree values for PPI networks generated for the AML vs T-ALL comparison

Node	TLR4	MPO	MNDA	CSF1R	CD44	C3AR1	FCER1G	CTSS	LYN	FOS
Description	down	down	down	down	down	down	down	down	down	down
Degree	32	28	26	26	25	23	23	23	21	20

Supplementary Table 17. The upregulated DEGs with the top 10 highest network degree values for PPI networks generated for the AML vs T-ALL comparison

Node	LAT	ITK	CD247	PRKCQ	TRAT1	RASGRP1	CD1B	CD2	DNTT	IL7R
Description	up	up	up	up	up	up	up	up	up	up
Degree	5	5	4	4	3	3	2	2	2	1

Supplementary Table 18. The downregulated DEGs with the top 10 highest network degree values for PPI networks generated for the AML vs T-ALL comparison

Node	TLR4	MPO	MNDA	CSF1R	C3AR1	CTSS	FCER1G	CD44	FOS	ELANE
Description	down	down	down	down	down	down	down	down	down	down
Degree	28	26	26	26	23	23	21	21	19	19

REFERENCES

1. Stone N, Kendall C, Smith J, Crow P, Barr H. Paul crow and hugh barr raman spectroscopy for identification of epithelial cancers. Faraday Discuss (2004)126: 141-57.

2. Pully VV, Lenferink A, Otto C. Time Lapse raman imaging of living cells. J Raman Spectrosc (2015) 42: 23.

3. González-Solís JL, Martínez-Espinosa JC, Salgado-Román JM, Palomares-Anda P. Monitoring of chemotherapy leukemia treatment using Raman spectroscopy and principal component analysis. Lasers Med Sci (2014) 29: 1241-9.

4. da Silva AM, de Siqueira E Oliveira F, de Brito P, Silveira L. Spectral model for diagnosis of acute leukemias in whole blood and plasma through Raman spectroscopy. J Biomed Opt (2018)23: 1-11.

5. Nargis HF, Nawaz H, Ditta A, Mahmood T, Majeed M, Rashid N, et al. Raman spectroscopy of blood plasma samples from breast cancer patients at different stages. Spectrochim Acta A Mol Biomol Spectrosc (2019) 222: 117210.

6. Managò S, Valente C, Mirabelli P, Circolo D, Basile F, Corda AC. A reliable Raman-spectroscopy-based approach for diagnosis, classification and follow-up of B-cell acute lymphoblastic leukemia. Sci Rep (2016) 6: 24821.
